# Complementary Syntheses Giving Access to a Full Suite of Differentially Substituted Phthalocyanine‐Porphyrin Hybrids

**DOI:** 10.1002/anie.202016596

**Published:** 2021-03-01

**Authors:** Faeza Alkorbi, Alejandro Díaz‐Moscoso, Jacob Gretton, Isabelle Chambrier, Graham J. Tizzard, Simon J. Coles, David L. Hughes, Andrew N. Cammidge

**Affiliations:** ^1^ School of Chemistry University of East Anglia Norwich Research Park Norwich NR4 7TJ UK; ^2^ UK National Crystallography Service Chemistry University of Southampton Southampton SO17 1BJ UK

**Keywords:** hybrids, phthalocyanines, porphyrinoids, selectivity

## Abstract

Phthalocyanines and porphyrins are often the scaffolds of choice for use in widespread applications. Synthetic advances allow bespoke derivatives to be made, tailoring their properties. The selective synthesis of unsymmetrical systems, particularly phthalocyanines, has remained a significant unmet challenge. Porphyrin‐phthalocyanine hybrids offer the potential to combine the favorable features of both parent structures, but again synthetic strategies are poorly developed. Here we demonstrate strategies that give straightforward, controlled access to differentially substituted meso‐aryl‐tetrabenzotriazaporphyrins by reaction between an aryl‐aminoisoindolene (A) initiator and a complementary phthalonitrile (B). The choice of precursors and reaction conditions allows selective preparation of 1:3 Ar‐ABBB and, uniquely, 2:2 Ar‐ABBA functionalized hybrids.

## Graphical abstract

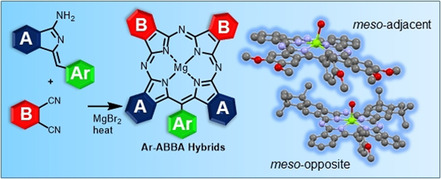

Phthalocyanines (Pc) and porphyrins are among the most widely studied functional organic materials. Porphyrin derivatives are widespread in nature and perform crucial life‐sustaining functions. Synthetic porphyrins and phthalocyanines are diversely used across chemical, biological and other advanced technology fields. Their popularity stems from a combination of general molecular properties such as light absorption and stability (a direct consequence of their extended aromaticity), and the ability to tune their physico‐chemical properties through a number of complementary strategies such as metal ion incorporation, perturbation of the core, and/or introduction of appropriate substituents.^[1]^

Hybrid structures, intermediate between Pc and porphyrins (Figure 1), were recognized as important scaffolds during the birth of Pc chemistry, and were discussed in Linstead's and Dent's original seminal series of papers in the 1930s.^[2]^ The hybrids possess complementary and superior characteristics to their parents, bridging the Pc and porphyrin systems and allowing precise tuning of their properties for specific applications.^[3]^ However, scarce synthetic availability of hybrid materials has limited the study of their scope. Synthetic procedures are mostly derived from the original methods from the 1930s, employing a carbon‐based nucleophile to initiate reaction with a phthalonitrile (Pn) co‐reactant.^[3]^ These strategies generally have poor yields and selectivity, leading most investigations to focus on hybrid structures bearing only simple or no substituents on the macrocycle or the *meso*‐carbon position. Interest in functional hybrids has been growing recently. We^[4]^ and others^[5]^ have refined C‐nucleophile procedures, extending studies to include substituents at the Pn and *meso*‐sites, and controlling product distribution through stoichiometry and reaction conditions. Access to the full range of (separable and processable) hybrids has further revealed their enhanced behavior as device components.^[6]^ More innovative synthetic inventions have recently started to redefine the field, charting the first steps towards controlled synthesis of di‐^[7]^ and triaza^[8]^ hybrids.

**Figure 1:**
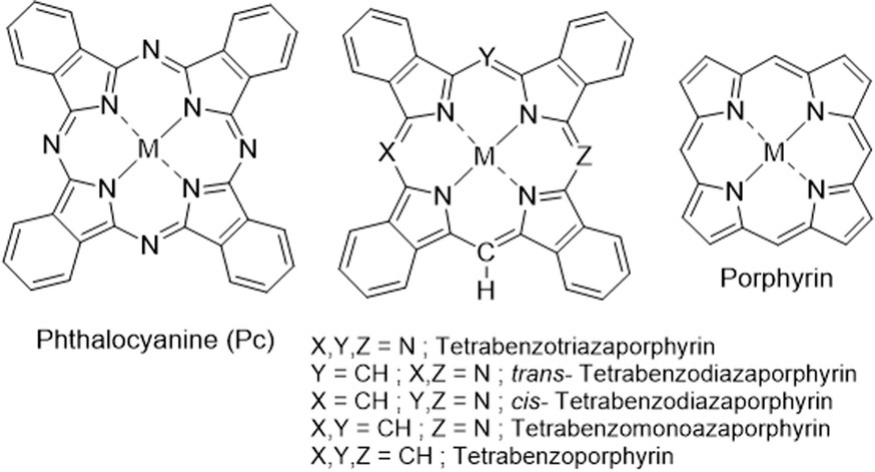
Molecular structures of phthalocyanine (Pc), porphyrin, and their hybrids.

Our synthesis of *meso*‐aryl tetrabenzotriazaporphyrins (TBTAPs) provided, for the first time, scalable access to these hybrid structures functionalized at the *meso* position.^[8]^ Based on the proposed mechanism, we recognized that our synthetic protocol had the potential to introduce different benzo fragments (**A** and **B**) around the macrocycle in a regiospecific manner, in addition to the *meso* functionality (Scheme 1). Such structural control has been long pursued in normal Pc chemistry with only limited advances.^[9]^ In the hybrid series, success would deliver materials that are unavailable in general Pc chemistry, but also offer the opportunity to further exploit the possibilities provided by the *meso*‐substituent.

**Scheme 1:**
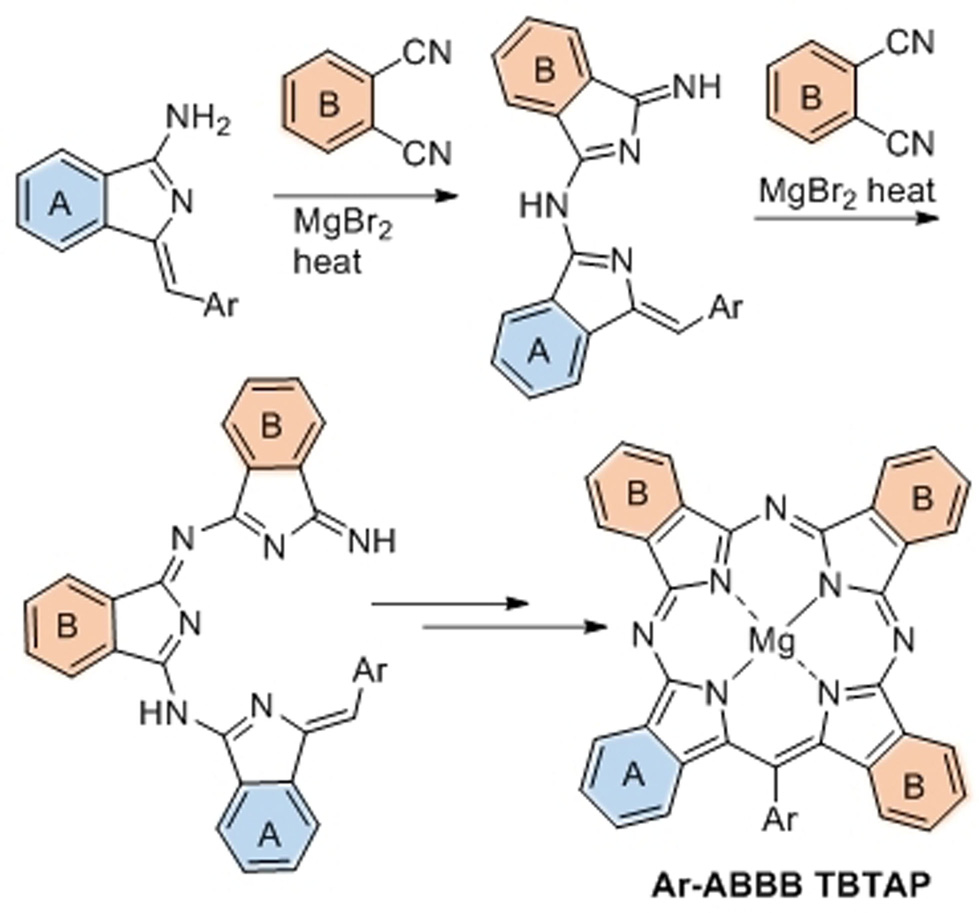
The synthesis of *meso*‐aryl TBTAPs from reaction between aminoisoindolene initiator^[10]^ (providing the A ring) and phthalonitrile (providing the B ring).^[8]^

Our first attempts to investigate the potential to introduce substituents onto TBTAP hybrids employed commercially available 4‐*tert*‐butylphthalonitrile,^[11]^ a widely used precursor in Pc chemistry that imparts good solubility to the final macrocycles. According to our proposed mechanism for this reaction, we expected to obtain a 1:3 peripheral substitution pattern (Scheme 1). However, reaction with our aminoisoindolene co‐reactant under the conditions optimized for TBTAP synthesis produced a complex mixture of products. Therefore, we shifted our strategy to symmetrically disubstituted Pns in order to simplify characterization and analysis. Several examples leading to peripheral substitution were chosen, avoiding steric clashes with substituents on the new *meso*‐carbon.[^4^, ^8^] Initial investigations used the Pn derivative **4**, derived from tetramethyl tetralin, synthesized from benzene by Friedel–Crafts alkylation,^[12]^ bromination,[^12^, ^13^] and cyanation.[^13b^, ^14^] An initial test reaction was performed using Pn **4** alone under the reaction conditions (MgBr_2_ in diglyme at reflux) to ensure that Pc formation did not occur directly at a competitive rate, as already shown for the unsubstituted phthalonitrile.^[8]^ TBTAP hybrid formation was then attempted following the previously optimized procedure, essentially by slowly adding aminoisoindolene “initiator” **6** to a mixture of Pn **4** (3–5 equiv) and MgBr_2_ in refluxing diglyme (Scheme 2).

**Scheme 2:**
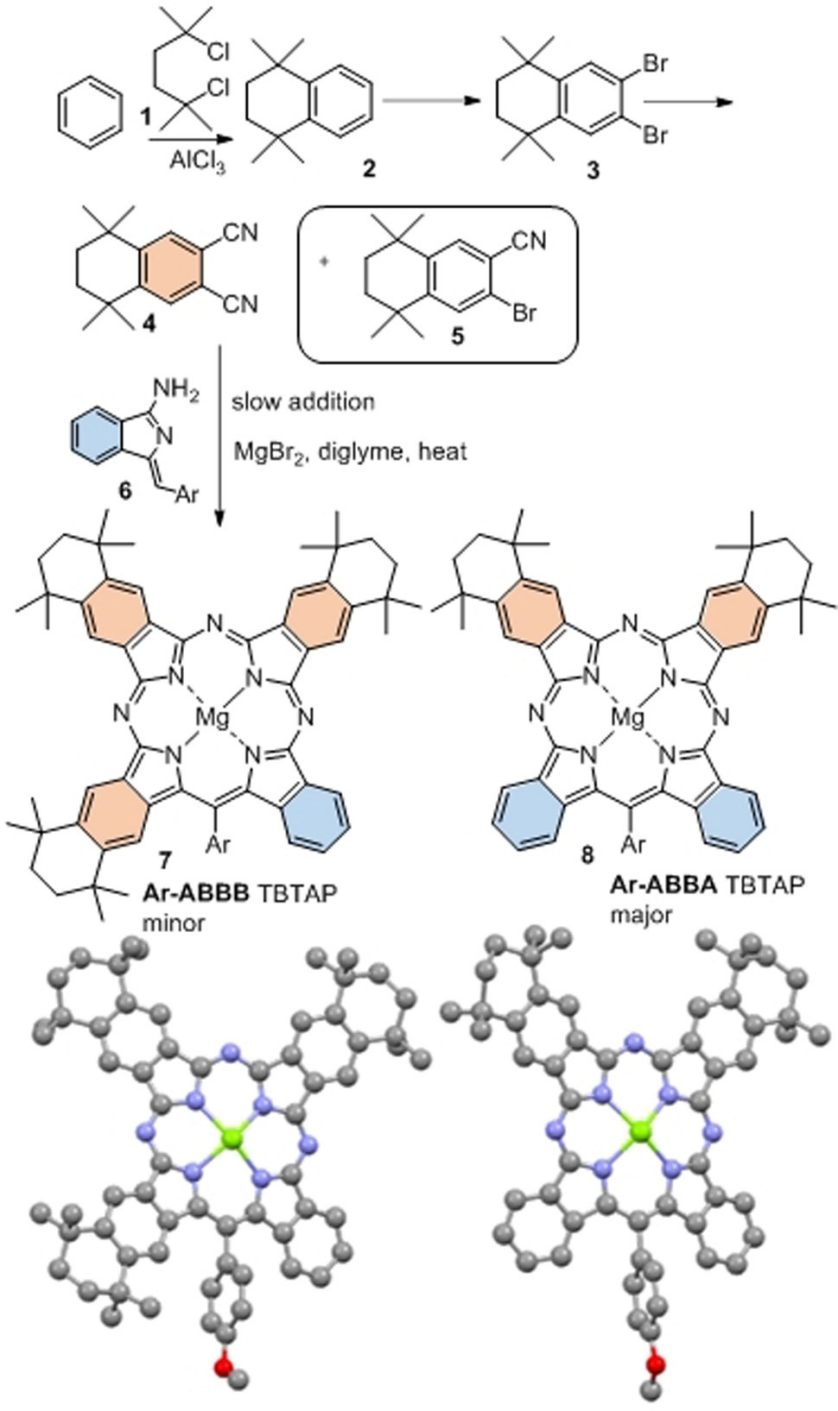
Hybrid synthesis from a substituted phthalonitrile, uncovering an alternative pathway leading to **Ar‐ABBA** TBTAP hybrid (Ar=4‐methoxyphenyl); crystal structures of hybrids **7** and **8** (solvent molecules omitted for clarity).

Macrocycle formation proceeded smoothly but two distinct hybrid products were isolated. The first product was characterized as the expected Ar‐ABBB (1:3) TBTAP hybrid **7** that likely results from the proposed sequential addition of aminoisoindolene to 3 Pn units, followed by cyclization and aromatization (Scheme 1). However, this component was the minor product. The dominant product was identified as the unique Ar‐ABBA (2:2) TBTAP hybrid **8**, produced as a single regioisomer. It is theoretically possible that this unexpected product is an artifact produced from the Ar‐ABBB hybrid **7** by a retro‐Friedel Crafts (de)alkylation under the reaction conditions. Although unlikely, this possibility was eliminated in a test experiment whereby Ar‐ABBB hybrid **7** was isolated and subjected to the reaction conditions (MgBr_2_ in refluxing diglyme). No reaction took place and it was therefore clear that the Ar‐ABBA hybrid **8** results from an alternative, dominant reaction sequence.

The most likely mechanism leading to hybrid **8** is shown in Scheme 3. It has the same first step as the mechanism proposed in Scheme 1, involving the initial addition of aminoisoindolene to Pn rendering an AB subunit (like all intermediates in Schemes 1 and 3, this is expected to be complexed to magnesium ion that is omitted for clarity), but the pathways then diverge. Addition of this intermediate to a second Pn eventually leads to the 1:3 ABBB hybrid but this appears to be a slow step. Self‐condensation of two AB intermediates (through loss of NH_3_) likely dominates, leading then to cyclization and aromatization via loss of a benzyl fragment. Of course, both pathways lead to the same product if unsubstituted Pn is employed as co‐reactant. Further support for this proposed sequence was provided by the results observed from changing the reaction stoichiometry and protocol. Switching to 2:2 aminoisoindolene:Pn stoichiometry and/or increasing the rate of addition (including reactions where all starting materials are mixed prior to heating) increased the relative proportion of Ar‐ABBA 2:2 TBTAP hybrid in the isolated macrocyclic product mixture. However, in such reactions the overall yield of both hybrids is reduced because self‐condensation of aminoisoindolene starts to compete with addition to Pn.

**Scheme 3:**
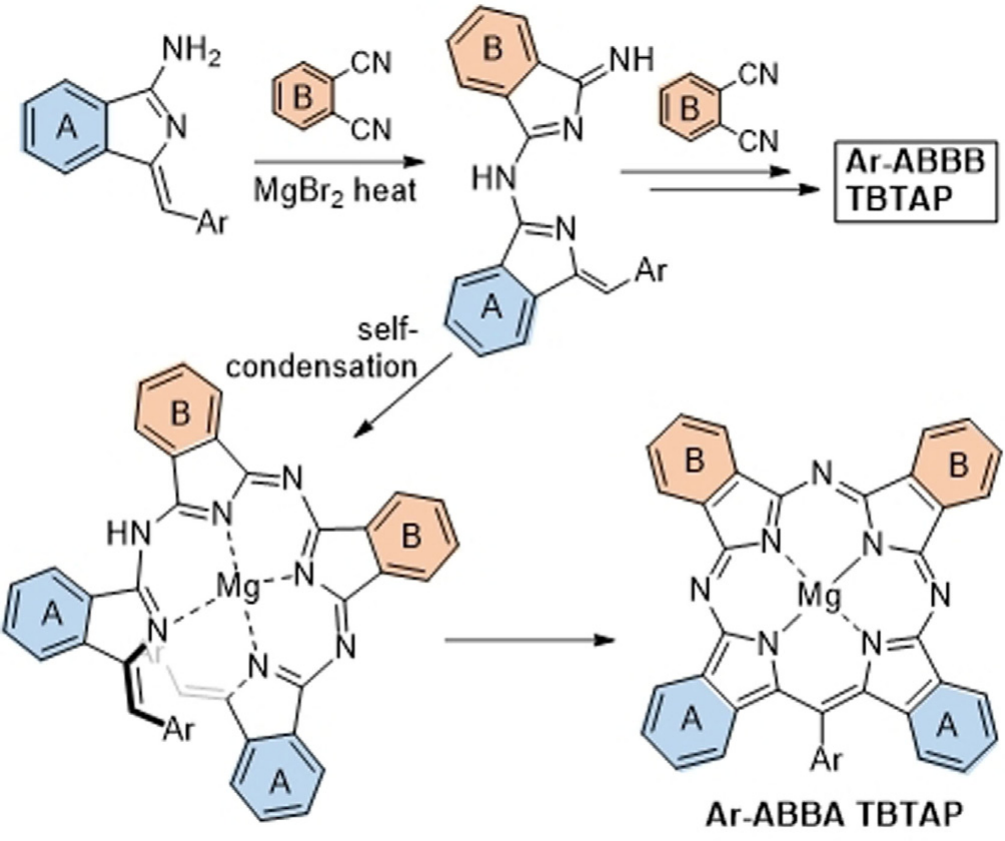
Proposed mechanistic pathway leading to **Ar‐ABBA** TBTAP hybrids.

The reaction, therefore, offers potential to produce two separate classes of TBTAP hybrids, both largely unprecedented. Two further series of experiments were carried out to demonstrate the scope. Firstly, alternative Pn derivatives were employed. 2,3‐Naphthalonitrile **9** is commercially available and underwent macrocyclization with aminoisoindolene **6** (Scheme 4). Naphthalonitrile **9** appears to be more reactive than Pn **4** and the reaction is complicated by competing formation of naphthalocyanine (MgNPc). Nevertheless, Ar‐ABBA TBTAP hybrid **10** was formed and isolated as the dominant hybrid once again. As expected, π‐extended mixed hybrid (*C*_2*v*_ symmetry) shows a split, red‐shifted Q‐band absorption at 709 and 681 nm.

**Scheme 4:**
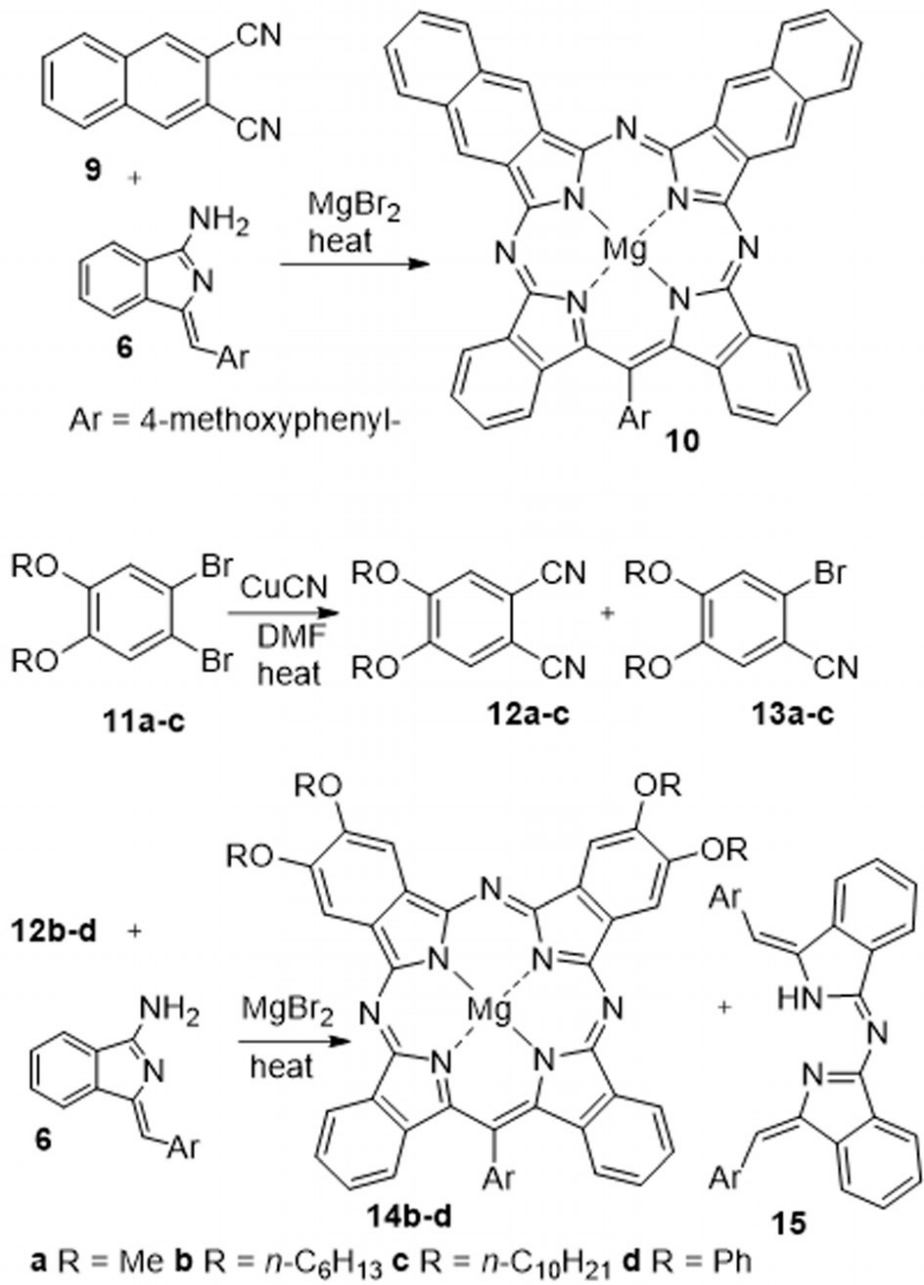
The synthesis of naphthyl (**10**, top) and tetraalkoxy/phenoxy (**14**, bottom) **Ar‐ABBA** TBTAP hybrids.

The second class of Pn selected were the 4,5‐dialkoxy derivatives **12**. In Pc chemistry these Pns are widely employed.^[15]^ They are known to be relatively easy to prepare at scale, and within the Pc series the alkoxy substituents modify the electronic, solubility and self‐assembly properties. The synthesis of the Pns and hybrids is shown in Scheme 4. Cyanation^[14]^ of dibromobenzene precursors **11** was carefully controlled to prevent excessive Pc formation under the reaction conditions. Stopping the reaction before completion resulted in a mixture of mono‐ (**13**) and dinitriles (**12**), but was a desirable outcome because we required the bromobenzonitrile derivatives for subsequent experiments (vide infra).

Dialkoxyphthalonitriles **12 a**–**c** were first shown not to react to form MgPcs in refluxing diglyme in the presence of MgBr_2_, allowing our standard reaction conditions to be employed for hybrid synthesis. Reaction of dimethoxy‐Pn **12 a** with aminoisoindolene, however, failed to produce significant quantities of macrocycle (hybrid or Pc) and instead yielded significant quantities of condensation product **15** (plus unreacted Pn), presumably due to solubility issues. Longer chain dihexyloxy‐ and didecyloxy‐Pn (**12 b** and **12 c**, and diphenoxy‐Pn (**12 d**, prepared from 4,5‐dichlorophthalonitrile), reacted smoothly, however, using the single‐operation procedure whereby a mixture of aminoisoindolene, Pn and MgBr_2_ were heated directly in diglyme. Once again, the dominant macrocyclic product isolated from these reactions was the Ar‐ABBA TBTAP. Separation proved to be challenging, but the pure Ar‐ABBA hybrids **14 b**–**d** could be isolated and characterized.

In all experiments described so far, an identical aminoisoindolene reactant was employed. Aminoisoindolene **6** has no substituents on the indolene fragment so delivers unsubstituted rings (“**A**”) into the Ar‐ABBA hybrids. Alternative substitution patterns become available if the indolene fragment is itself functionalized, and in such cases the syntheses will deliver hybrids with substituents on the rings adjacent to the *meso*‐Ar. This complementary sequence has been demonstrated using the precursor bromobenzonitriles prepared as part of the earlier Pn synthesis, and is indeed a powerful approach (Scheme 5).

**Scheme 5:**
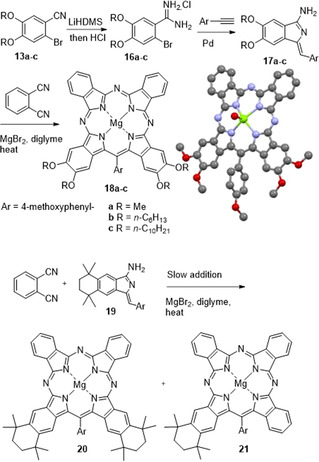
Complementary synthesis of TBTAP hybrids to introduce substituents adjacent to the *meso*‐carbon.

Bromobenzonitriles **13 a**–**c** were converted to the corresponding amidine hydrochloride salts (**16 a**–**c**) by treatment with LiHDMS followed by HCl workup.^[16]^ The amidines were converted to aminoisoindolenes (**17 a**–**c**) by reaction with 4‐methoxyphenyl acetylene under palladium catalysis.^[17]^ In accordance with our previous results, reaction of these substituted aminoisoindolenes with phthalonitrile in a single operation led to formation of the complementary (*meso*‐adjacent) Ar‐ABBA TBTAP hybrids **18 a**–**c** as the dominant macrocyclic products, alongside traces of the 1:3 Ar‐ABBB TBTAP and phthalocyanine (identified by MALDI‐MS). Ar‐ABBA hybrids **18 a**–**c** were isolated pure by chromatography and recrystallization. In the case of the methoxy‐substituted TBTAP **18 a**, crystals suitable for X‐ray diffraction were obtained and the crystal structure is also shown in Scheme 5.

Unlike the dialkoxy derivatives, aminoisoindolene **19** (prepared from bromobenzonitrile **5** by the same reaction sequence described for **17**) is freely soluble in diglyme enabling the reaction to be performed by slow addition (syringe pump) to phthalonitrile and therefore allowing the sequential addition mechanism to compete more effectively. Under these conditions the 1:3 TBTAP hybrid **21** could indeed also be isolated, although it remains a minor component compared to the Ar‐ABBA 2:2 hybrid **20**. This effectively completes the series and demonstrates that the full suite of hybrid structures can be accessed at will (Figure 2).

**Figure 2:**
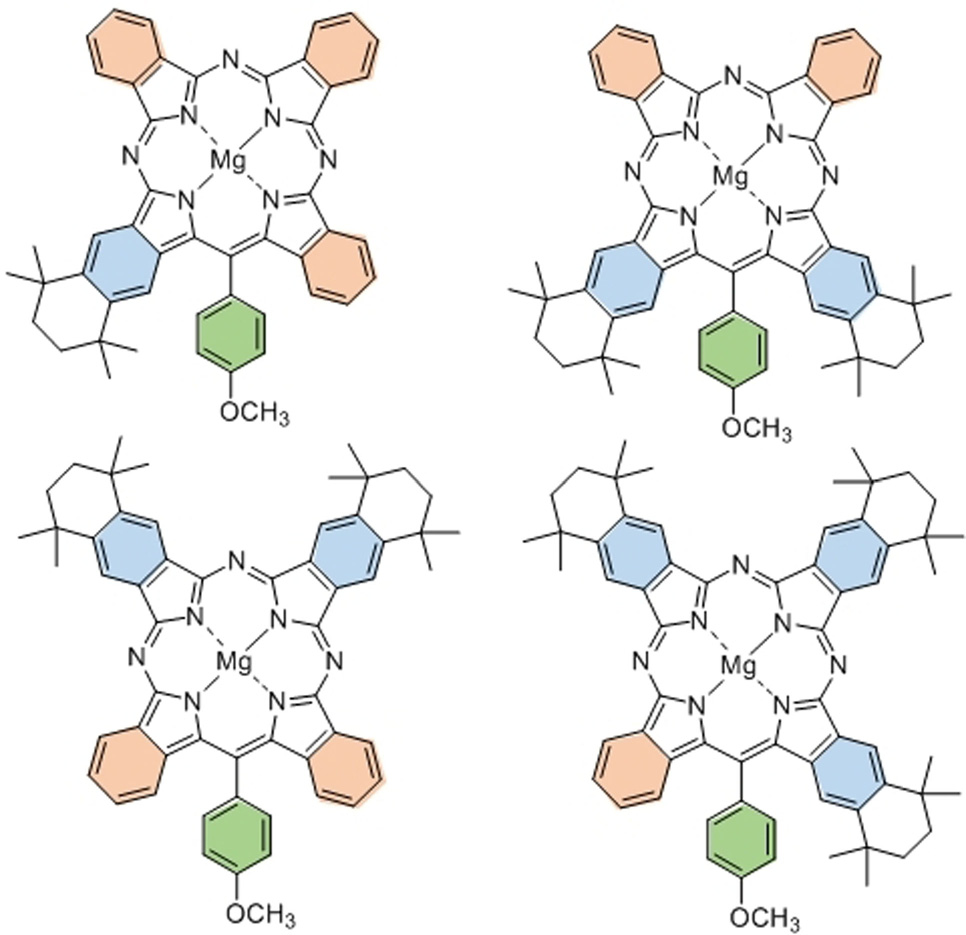
Suite of TBTAP hybrids that can now be synthesized controlling the *meso*‐aryl, adjacent, and opposite benzo‐substituents.

In conclusion, two pathways are proposed for the synthesis of an important class of functionalized phthalocyanine‐porphyrin hybrids (TBTAPs). The materials are novel in their own right, but more importantly, the syntheses offer control and variation over structural and substituent modifications, a goal not yet achieved even within the extensively investigated chemistry of the parent phthalocyanines. Differential substitution can be controlled leading to a full range of complementary functionality, at the *meso*‐carbon itself (ideal for attachment of these functional antennae^[18]^ molecules) and at one or both of the adjacent or opposite benzo sites to the *meso*‐carbon (controlling molecular electronic character but also permitting design of super‐ and supramolecular functional assemblies^[19]^).

## Conflict of interest

The authors declare no conflict of interest.

## Supporting information

As a service to our authors and readers, this journal provides supporting information supplied by the authors. Such materials are peer reviewed and may be re‐organized for online delivery, but are not copy‐edited or typeset. Technical support issues arising from supporting information (other than missing files) should be addressed to the authors.

Supplementary
