# Supplementary material for: Complementary Syntheses Giving Access to a Full Suite of Differentially Substituted Phthalocyanine‐Porphyrin Hybrids
Source: Angew Chem Int Ed Engl. 2021 Mar 1;60(14):7632–6. doi: 10.1002/anie.202016596 (PMC8048519; doi:10.1002/anie.202016596)
Supplement: Supplementary file 1 — Supplementary [file ANIE-60-7632-s001.pdf]

Angewandte Chemie
Eine Zeitschrift der Gesellschaft Deutscher Chemiker

Supporting Information

Complementary Syntheses Giving Access to a Full Suite of Differentially Substituted Phthalocyanine-Porphyrin Hybrids

Faeza Alkorbi, Alejandro Díaz-Moscoso, Jacob Gretton, Isabelle Chambrier, Graham J. Tizzard, Simon J. Coles, David L. Hughes, and Andrew N. Cammidge**

anie_202016596_sm_miscellaneous_information.pdf

Supporting Information

Contents

Syntheses and characterisation details……………………………………..2

Cyanation reactions…………………………………………………………………3

Amidines……………………………………………………………………………..6

Aminoisoindolenes…………………………………………………………………..9

General synthesis of TBTAP hybrids…………………………….……………..15

“Opposite” series……………………………………………………………………16

“Adjacent” Series……………………………………………………….……..26

Crystallographic details…………………………………….……….....35

References………………………………………………………….…..44

Synthesis and characterisation details

General Methods

Reagents and solvents were obtained from commercial sources and used without further purification unless otherwise stated. Phthalonitrile was recrystallised from hot xylene. THF was freshly distilled from sodium and benzophenone. Reactions and distillation were carried out under an inert atmosphere (argon or nitrogen gas), in most air-sensitive reactions argon was preferred. Brine is a saturated aqueous solution of sodium chloride. Organic layers were dried using anhydrous magnesium sulphate. Evaporating of solvent was performed using a Buchi rotary evaporator at reduced pressure. ^{1}H NMR spectra were recorded either at 400 MHz on Ultrashield Plus^{TM} 400 spectrometer or 500 MHz on a Bruker Ascend^{TM} 500 spectrometer in 5 mm diameter tubes. Signals are quoted in ppm as δ downfield from tetramethylsilane (δ=0.00) and coupling constants *J* given in Hertz. ^{13}C[^{1}H] NMR spectra were recorded at 100.6 MHz or 125.7 MHz on the same spectrometers. NMR spectra were performed in solution using deuterated chloroform, methanol, dichloromethane or tetrahydrofuran at room temperature unless otherwise stated. Ultraviolet-Visible absorption spectra were recorded on Hitachi U-3310 Spectrophotometer in solvent as stated. MALDI-TOF mass spectra were carried out using a Shimadzu Biotech Axima instrument. Characterization of hybrids by MALDI-tof mass spectrometry was achieved by comparison of isotopic distribution to theory. IR spectra were recorded using a Perkin-Elmer Spectrum BX FT-IR spectrometer. Thin layer chromatography (TLC) was performed using aluminium sheets coated with Alugram® Sil G/UV254 (Macherey-Nagel), and the compounds were visualised under short-wavelength UV-light at 245 nm or 366 nm. Column chromatography was carried out using silica gel 60Å mesh 70-230 (63-200 µm) under gravity or moderate pressure at ambient temperature. Solvent ratios are given as v:v. Melting points were taken on a Reichart Thermovar microscope with a thermopar based temperature control. Reactions using microwave irradiation were carried out in Biotage Initiator+ Microwave system.

The following compounds were prepared as previously described in literature: 2,5-dichloro-2,5-dimethylhexane **1**,[1] 1,1,4,4-tetramethyl-1,2,3,4-tetrahydro-naphthalene **2**,[2] 6,7-dibromo-1,2,3,4-tetrahydro-1,1,4,4-tetramethylnaphthalene **3**,[3] 1,2-dibromo-4,5-dimethoxybenzene **11a**,[4] 1,2-dibromo-4,5-bis(hexyloxy)benzene **11b**,[5] 1,2-dibromo-4,5-bis(decyloxy) benzene **11c**.[6]

General cyanation reaction conditions (Rosenmund–von Braun synthesis)[7]

A mixture of 1,2-dibromo-benzene derivative (4.39 mmol) and CuCN (1.97 g, 21.99 mmol) was refluxed in dry DMF (15 ml) under an argon atmosphere and the reaction was monitored by TLC. After ca. 3 hours, the reaction mixture was cooled to room temperature, DCM (20 ml) was added and then the mixture was filtered to remove the copper salts. The filtrate was washed with water (3 x 50 mL) and with an aqueous solution of ammonia until no blue colour was obtained in the aqueous layer. Finally, the organic layer was washed with a saturated solution of $NaHCO_3$, dried over $MgSO_4$ and filtered. The filtrate was evaporated under reduced pressure to give the crude product. The crude material was purified by column chromatography (silica: (1:2) DCM:PE → (1:1) DCM:PE → DCM) to yield mono- and dinitriles which are both isolated from the column.

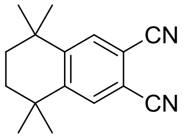

6,7-Dicyano-1,2,3,4-tetrahydro-1,1,4,4-tetramethylnaphthalene (4)

This compound was synthesised from **3** following the general cyanation reaction procedure to give a yellow solid (0.34 g, 33%). **M.p.** 204-205°C (lit. 206-208°C).[8] **^{1}H NMR** (500 MHz, $CDCl_3$): δ = 7.71 (s, 2H), 1.72 (s, 4H), 1.30 (s, 12H). **^{13}C NMR** (125.7 MHz, $CDCl_3$): δ = 151.80, 132.62, 116.05, 112.58, 35.13, 34.10, 31.45. **FT-IR** (NaCl): ῦ (cm^{-1}) = 2232 (C≡N).

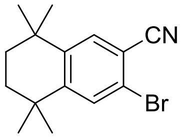

6-Bromo-7-cyano-1,2,3,4-tetrahydro-1,1,4,4-tetramethylnaphthalene (5)

This compound was isolated as side product from cyanation reaction of **3** (0.57 g, 45%). **M.p.** 158-159°C. **^{1}H NMR** (500 MHz, $CDCl_3$): δ = 7.57 (s, 1H), 7.55 (s, 1H), 1.68 (s, 4H), 1.27 (s, 6H), 1.27 (s, 6H). **^{13}C NMR** (125.7 MHz, $CDCl_3$): δ = 152.82, 145.53, 133.29, 131.41, 121.53, 117.87, 112.96, 35.13, 34.45, 34.41, 31.67, 31.54. **FT-IR** (NaCl): ῦ (cm^{-1}) = 2232 (C≡N).

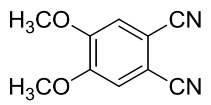

1,2-Dicyano-4,5-dimethoxybenzene (12a)

This compound was synthesised from **11a** following the general cyanation reaction to give a colorless solid (0.26 g, 32%). **M.p.** 182-184°C (lit. 179-181°C).[9] **^{1}H NMR** (500 MHz, $CDCl_3$): δ = 7.15 (s, 2H), 3.97 (s, 6H, OCH_3). **^{13}C NMR** (100.6 MHz, $CDCl_3$): δ = 152.73, 115.86, 114.90, 109.12, 56.79 (OCH_3). **FT-IR** (NaCl): ῡ (cm^{-1}) = 2225 (C≡N).

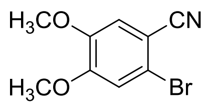

2-Bromo-4,5-dimethoxybenzonitrile (13a)

This compound was isolated as side product from the cyanation of **11a** (0.45 g, 42%). **M.p.** 118-120°C (lit. 113°C).[10] **^{1}H NMR** (400 MHz, $CDCl_3$): δ = 7.07 (s, 1H,), 7.05 (s, 1H), 3.93 (s, 3H), 3.89 (s, 3H). **^{13}C NMR** (100.6 MHz, $CDCl_3$): δ = 153.33, 148.65, 117.76, 117.73, 115.63, 115.39, 107.03, 56.62, 56.50. **FT-IR** (NaCl): ῡ (cm^{-1}) = 2230 (C≡N).

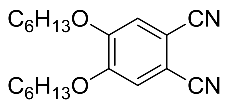

1,2-Dicyano-4,5-bis(hexyloxy)benzene (12b)

This compound was synthesised from **11b** following the general cyanation reaction to give a colorless solid (1.05 g, 36%). **M.p.** 104.6-106°C (lit. 100.3°C).[6a] **^{1}H NMR** (500 MHz, $CDCl_3$): δ = 7.11 (s, 2H), 4.05 (t, 4H), 1.87-1.82 (m, 4H), 1.50-1.44 (m, 4H), 1.38-1.30 (m, 8H), 0.92-0.89 (m, 6H). **^{13}C NMR** (125.7 MHz, $CDCl_3$): δ = 152.60, 116.10, 115.91, 108.50, 69.85, 31.51, 28.79, 25.61, 22.65, 14.08. **FT-IR** (NaCl): ῡ (cm^{-1}) = 2228 (C≡N).

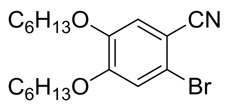

2-Bromo-4,5-bis(hexyloxy)benzonitrile (13b)

This compound was isolated as an additional product from cyanation of **11b** (1.94 g, 58%). **M.p.** 52.6°C. **^{1}H NMR** (500 MHz, $CDCl_3$): δ = 7.04 (s, 1H), 7.03 (s, 1H), 4.01 (t, 2H), 3.96 (t, 2H) 1.86-1.78 (m, 4H), 1.49-1.43 (m, 4H), 1.36-1.32 (m, 8H), 0.93-0.88 (m, 6H). **^{13}C NMR** (125.7 MHz, $CDCl_3$): δ = 153.57, 148.43, 117.98, 117.42, 117.24, 116.75, 106.57, 69.80, 69.64, 31.61, 31.56, 29.02, 28.90, 25.70, 25.67, 22.70, 22.68, 14.13, 14.11. **FT-IR** (NaCl): ṽ (cm^{-1}) = 2231 (C≡N).

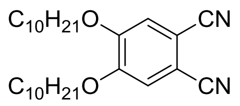

1,2-Dicyano-4,5-bis(decyloxy)benzene (12c)

This compound was synthesised from **11c** following the general cyanation reaction to give a colorless solid (1.1 g, 38%). **M.p.** 100-102°C (lit. 106°C).[6b] **^{1}H NMR** (500 MHz, $CDCl_3$): δ = 7.11 (s, 2H), 4.04 (t, *J* = 6.5 Hz, 4H), 1.84 (m, 4H), 1.52-1.42 (m, 4H), 1.39-1.16 (m, 24H), 0.88 (t, *J* = 6.8 Hz, 6H). **^{13}C NMR** (125.7 MHz, $CDCl_3$): δ = 152.61, 116.10, 115.93, 108.53, 69.87, 32.03, 29.68, 29.66, 29.46, 29.38, 28.85, 25.96, 22.82, 14.25. **FT-IR** (NaCl): ṽ (cm^{-1}) = 2229 (C≡N).

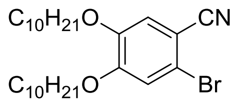

2-Bromo-4,5-bis(decyloxy)benzonitrile (13c)

This compound was isolated as an additional product from cyanation of **11c** (1.85 g, 57%). **M.p.** 80-81°C. **^{1}H NMR** (500 MHz, $CDCl_3$): δ = 7.03 (s, 2H), 4.01 (t, *J* = 6.6 Hz, 2H), 3.96 (t, *J* = 6.5 Hz, 2H), 1.86-1.78 (m, 4H), 1.53-1.46 (m, 4H), 1.40-1.19 (m, 24H), 0.89-0.86 (m, 6H). **^{13}C NMR** (100.6 MHz, $CDCl_3$): δ = 153.59, 148.45, 117.98, 117.42, 117.29, 116.79, 106.58, 69.82, 69.65, 32.05, 29.72, 29.69, 29.47, 29.46, 29.42, 29.07, 28.95, 26.04, 26.00, 22.83, 14.26. **FT-IR** (NaCl): ṽ (cm^{-1}) = 2230 (C≡N).

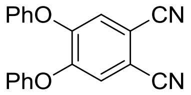

1,2-Dicyano-4,5-bis(phenoxy)benzene (12d)

A mixture of 4,5-dichlorophthalonitrile (1.0 g, 5.1 mmol), phenol (1.4 g,15.3 mmol), K_2CO_3 (3.0 g, 22 mmol) and anhydrous DMF (30 ml) was stirred at 110°C for 8 h, cooled, and diluted with 100 ml of water. The precipitate was filtered off, washed with 5% aqueous KOH and with water to pH 7, and dried at 70°C. The solid was recrystallized from methanol to afford the product as off-white crystals[11] (1.3 g, 81%). **M.p.** 140-145°C (lit. 149-150°C).[4b] **^{1}H NMR** (500 MHz, $CDCl_3$): δ = 7.46 (t, J = 7.9 Hz, 4H), 7.30 (t, J = 7.4 Hz, 2H), 7.17 (s, 2H), 7.09 (d, J = 8.0 Hz, 4H). **^{13}C NMR** (125.7 MHz, $CDCl_3$): δ = 154.23, 152.08, 130.71, 126.10, 122.09, 120.04, 115.14, 110.48. **FT-IR** (NaCl): ῦ (cm^{-1}) = 2236 (C≡N).

General synthesis of 2-bromobenzamidine hydrochlorides

Following the method reported by Dalai *et al.*,[12] a solution of 2-bromobenzonitrile derivative (20.33 mmol) in dry THF (3 ml) was added to a solution of $LiN(SiMe_3)_2$ in anhydrous THF (1M, 22 ml, 22 mmol, 1.1eq). The reaction mixture was stirred at room temperature for 4 h. A 5N HCl solution in isopropanol (15 ml) was added to the cooled mixture. The crude reaction mixture was left to stir at room temperature overnight. The precipitate was filtered off and washed with diethyl ether to give the title compound which was then recrystallized from methanol.

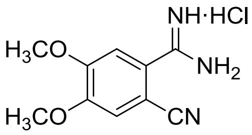

2-Bromo-4,5-dimethoxybenzamidine hydrochloride (16a)

Synthesised following the general procedure for amidines using 2-bromo-4,5-dimethoxybenzonitrile **13a** to yield **16a** as colorless crystals (2.5 g, 89%). **M.p.** 148-150°C. **^{1}H NMR** (500 MHz, CD_3OD): δ = 7.30 (s, 1H), 7.19 (s, 1H), 3.90 (s, 3H, OCH_3), 3.88 (s, 3H, OCH_3). **^{13}C NMR** (125.7 MHz, CD_3OD): δ = 167.97, 153.86, 150.28, 124.3, 117.42, 113.64, 112.03, 57.0, 56.97.

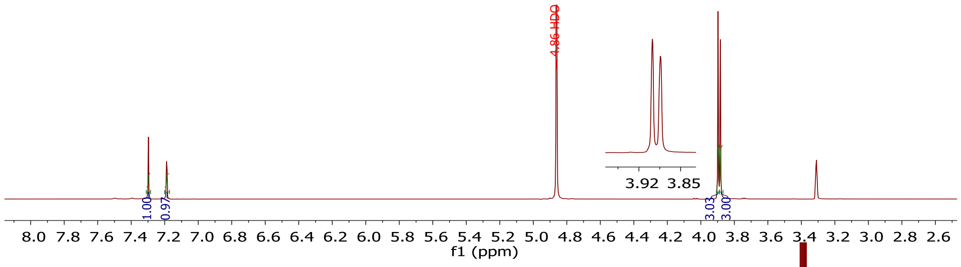

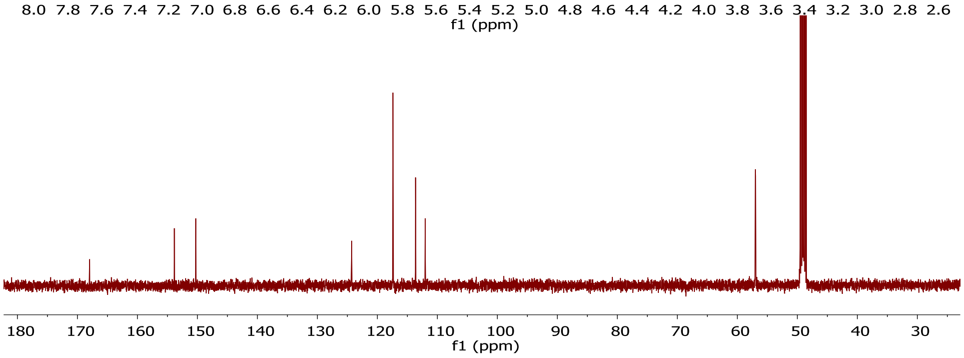

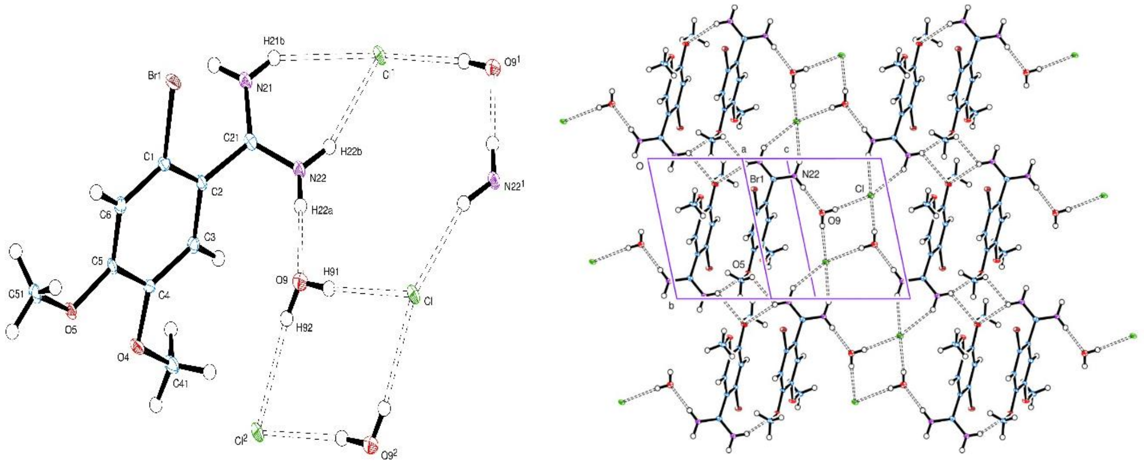

(full crystallographic details below)

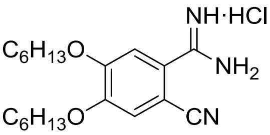

2-Bromo-4,5-bis(hexyloxy)-benzamidine hydrochloride (16b)

Synthesised following the general synthesis of amidines using **13b** to yield **16b** as colorless crystals (3.9 g, 82%). **M.p.** 158.9-160°C. **^{1}H NMR** (500 MHz, CD_3OD): δ = 7.27 (s, 1H), 7.14 (s, 1H), 4.07-4.02 (m, 4H), 1.83-1.78 (m, 4H), 1.54-1.48 (m, 4H), 1.39-1.35 (m, 8H), 0.94-0.91 (m, 6H). **^{13}C NMR** (125.7 MHz, CD_3OD): δ = 168.04, 153.80, 150.00, 124.18, 118.66, 115.18, 111.88, 70.70, 70.57, 32.69, 30.21, 30.10, 26.86, 26.82, 23.70, 14.39.

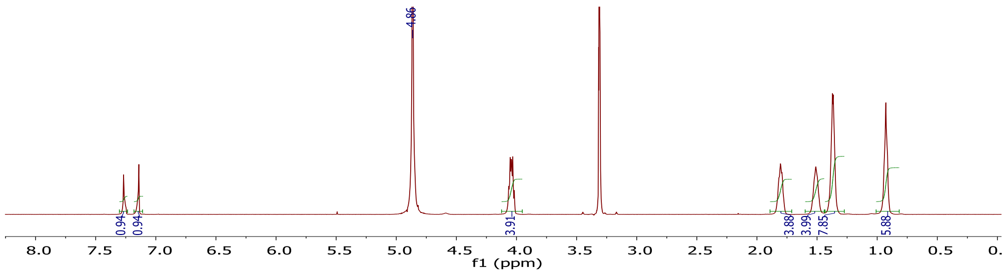

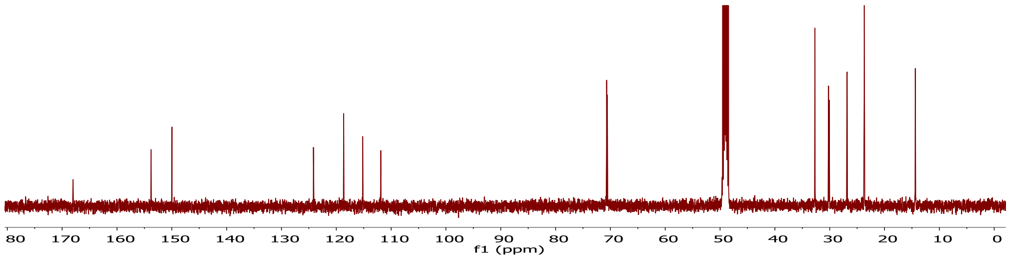

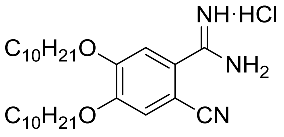

2-Bromo-4,5-bis(decyloxy)-benzamidine hydrochloride (16c)

Synthesised following the general procedure for amidines using **13c** to give **16c** as colorless crystals (4.68 g, 61%). **M.p.** 159-161°C. ^{1}**H NMR** (500 MHz, CD_3OD): δ = 7.27 (s, 1H), 7.15 (s, 1H), 4.07-4.02 (m, 4H), 1.84-1.78 (m, 4H), 1.54-1.48 (m, 4H), 1.40-1.31 (m, 24H), 0.92-0.89 (m, 6H). ^{13}**C NMR** (125.7 MHz, CD_3OD): δ = 168.04, 153.82, 150.01, 124.18, 118.68, 115.21, 111.89, 70.71, 70.57, 33.11, 30.83, 30.82, 30.73, 30.52, 30.50, 30.28, 30.16, 27.24, 27.20, 23.77, 14.47.

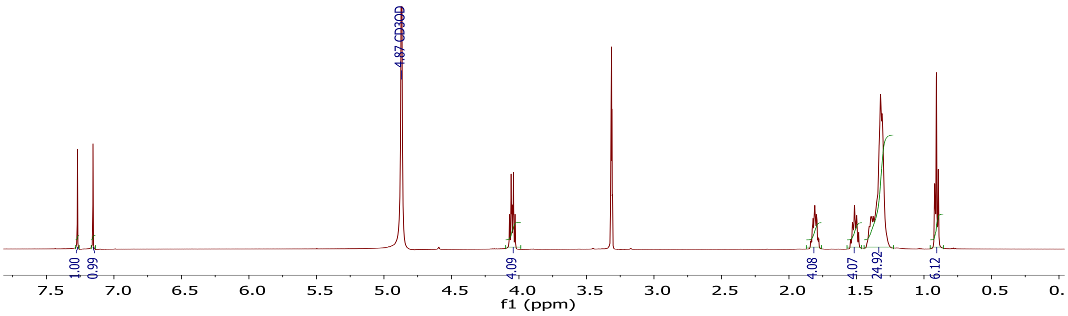

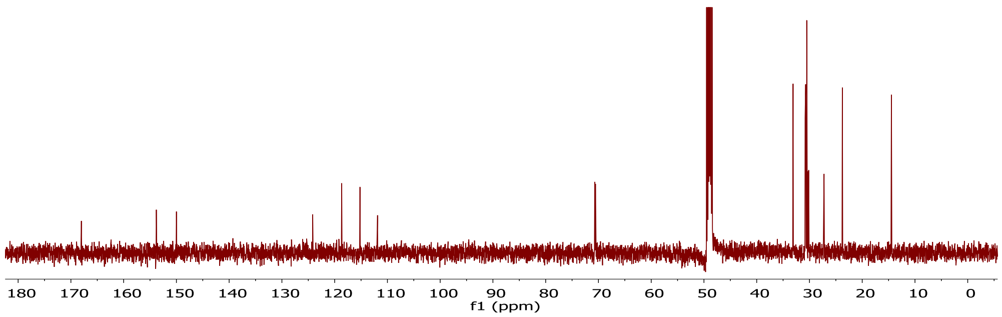

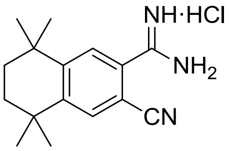

6-Bromo-1,1,4,4-tetramethyl-1,2,3,4-tetrahydronaphthalene-7-imidamide hydrochloride (22)

Synthesised following the general procedure for amidines using 6-bromo-7-cyano-1,2,3,4-tetrahydro-1,1,4,4-tetramethylnaphthalene **5** to yield **22** as colorless crystals (1.23 g, 77%). **M.p.** 153-155°C. **^{1}H NMR** (500 MHz, CD_3OD): δ = 7.70 (s, 1H), 7.56 (s, 1H), 1.75 (s, 4H), 1.33 (s, 6H), 1.31 (s, 6H). **^{13}C NMR** (125.7 MHz, CD_3OD): δ = 168.28, 152.68, 152.66, 146.72, 132.82, 130.23, 129.37, 117.45, 35.72, 35.56, 35.52, 35.35, 31.80, 31.79.

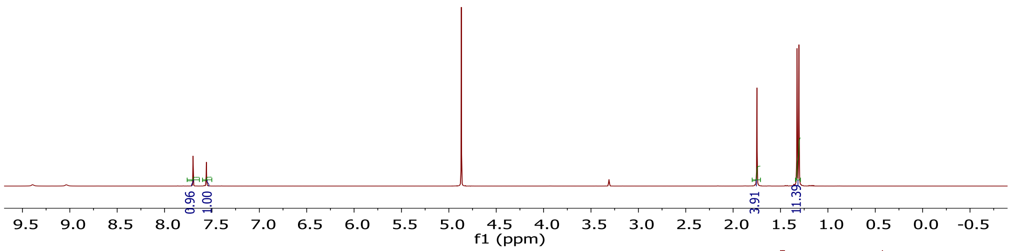

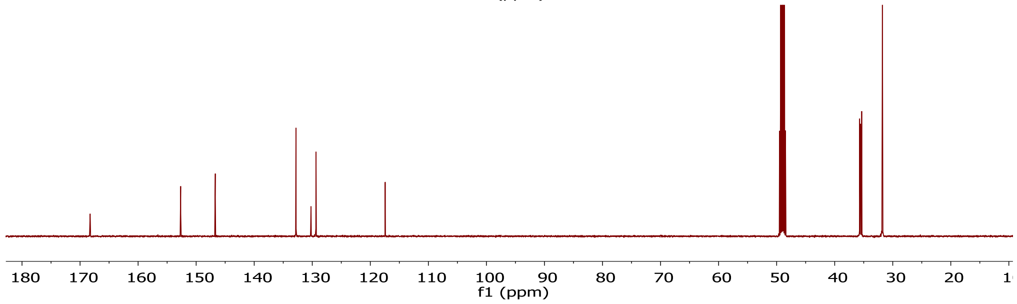

<u>General synthesis of aminoisoindolenes</u>[13]

A mixture of 2-bromobenzamidine hydrochloride (3 mmol), BINAP (102 mg, 0.165 mmol, 0.055eq) and $PdCl_2(MeCN)_2$ (39 mg, 0.15 mmol, 0.05eq) was sealed in a microwave vessel with a magnetic bar and then purged and refilled with N_2 three times. Then, 4-ethynylanisole (0.467 ml, 0.476 g, 3.6 mmol, 1.2eq) and DBU (1.12 ml, 1.14 g, 7.5 mmol, 2.5eq) in dry DMF (12 ml) were added. The mixture was stirred under N_2 for 5 min to give a clear yellow solution with a white solid. The mixture was irradiated in a microwave reactor at 120°C for 1 h. After cooling, ethyl acetate (50 ml) was added and the mixture washed with a saturated solution of $NaHCO_3$ (3x75 ml). The organic layer was dried with $MgSO_4$, filtered and concentrated. The residue was finally purified by column chromatography using PE:AcOEt (1:1) and then AcOEt as solvent gradient to afford a yellow semisolid that was recrystallized from DCM:PE (1:1) to yield the corresponding aminoisoindolenes.

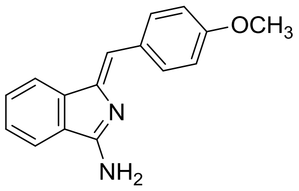

(*Z*)-1-[(4-Methoxy)benzylidene]-1*H*-isoindol-3-amine (6)[14]

Following the general procedure for synthesis of aminoisoindolenes, aminoisoindolene **6** was isolated as yellow needles (620 mg, 83%). **^{1}H NMR** (400 MHz, $CDCl_3$): δ = 7.99 (d, *J* = 8.7 Hz, 2H), 7.77 (d, *J* = 7.8 Hz, 1H), 7.68 (d, *J* = 7.7 Hz, 1H), 7.50 (td, *J* = 7.5, 1.0 Hz, 1H), 7.38 (td, *J* = 7.5, 0.9 Hz, 1H), 6.94 (d, *J* = 8.7 Hz, 2H), 6.76 (s, 1H), 3.83 (s, 3H). **^{13}C NMR** (100.6 MHz, $CDCl_3$): δ = 164.05, 159.56, 142.60, 142.07, 132.00, 130.11, 129.51, 128.77, 127.43, 120.26, 119.78, 115.60, 114.36, 55.46. **MS (MALDI-TOF):** *m/z* = 250.1 $[M]^+$ (100%). **UV-vis** (DCM): λ_{max} (nm) (ε ($dm^3 \cdot mol^{-1} \cdot cm^{-1}$)) = 372 ($3.33 \cdot 10^4$).

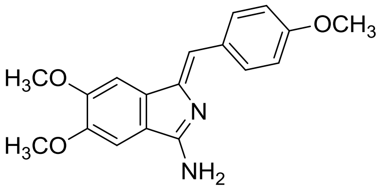

(*Z*)-5,6-Dimethoxy-1-[(4-methoxy)benzylidene]-1*H*-isoindol-3-amine (17a)

Synthesised from 2-bromo-4,5-dimethoxybenzamidine hydrochloride **16a** following the general aminoisoindolene synthesis, giving **17a** as yellow needles (382 mg, 41%). **M.p.** 220-223°C. **^{1}H NMR** (500 MHz, $CDCl_3$): δ = 7.99 (d, *J* = 8.8 Hz, 2H), 7.23 (s, 1H), 7.02 (s, 1H), 6.93 (d, *J* = 8.8 Hz, 2H), 6.59 (s, 1H), 4.02 (s, 3H), 3.94 (s, 3H), 3.83 (s, 3H). **^{13}C NMR** (125.7 MHz, $CDCl_3$): δ = 164.33, 159.24, 151.66, 149.56, 143.65, 136.40, 131.70, 129.25, 122.84, 114.92, 114.27, 102.18, 102.08, 56.48, 56.39, 55.45. **MS (MALDI-TOF):** *m/z* = 310.98 $[M]^+$ (100%). **UV-vis** (DCM): λ_{max} (nm) (ε ($dm^3 \cdot mol^{-1} \cdot cm^{-1}$)) = 362 ($2.15 \cdot 10^4$).

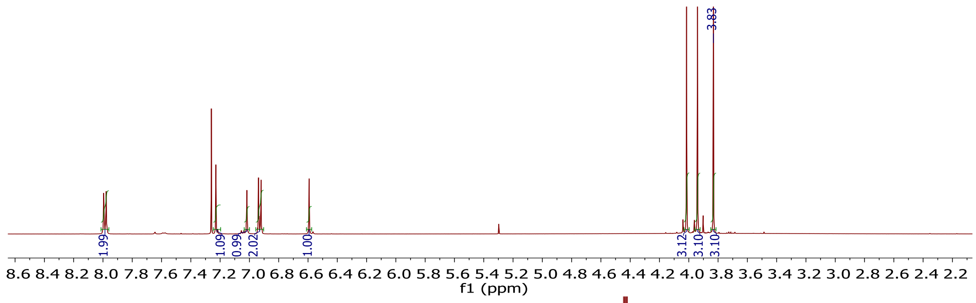

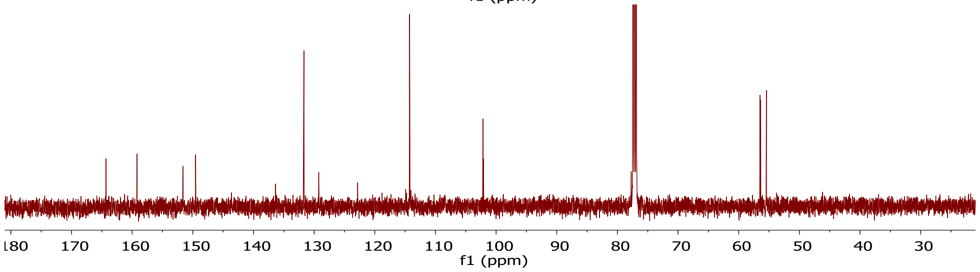

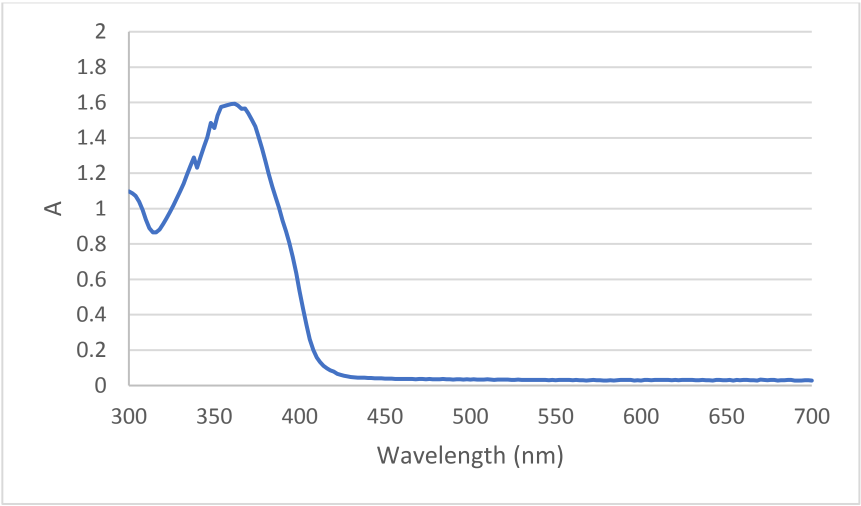

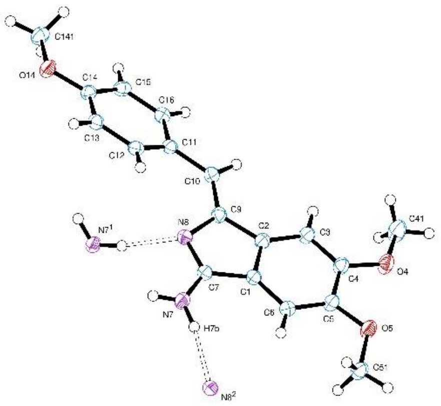

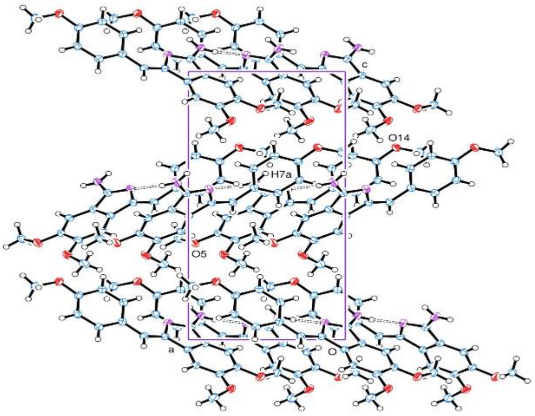

(further crystallographic details below)

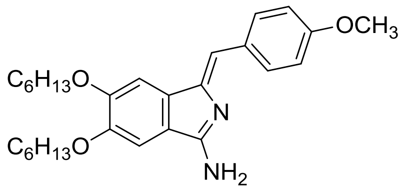

(*Z*)-5,6-bis(hexyloxy)-1-[(4-methoxy)benzylidene]-1*H*-isoindol-3-amine (17b)

Synthesised from 2-bromo-4,5-bis(hexyloxy)-benzamidine hydrochloride **16b** following the general aminoisoindolene synthesis giving **17b** as yellow needles (450 mg, 32%). **M.p.** 147-150°C. **^{1}H NMR** (500 MHz, $CDCl_3$): δ = 8.00 (d, *J*= 8.8 Hz, 2H), 7.22 (s, 1H), 6.95-6.87 (m, 3H), 6.55 (s, 1H), 4.11 (t, *J* = 6.6 Hz, 2H), 3.99 (t, *J* = 6.6 Hz, 2H), 3.82 (s, 3H), 1.91-1.81 (m, 4H), 1.54-1.46 (m, 4H), 1.38-1.33 (m, 8H), 0.94-0.85 (m, 6H). **^{13}C NMR** (125.7 MHz, $CDCl_3$): δ = 164.95, 158.94, 151.51, 149.36, 145.31, 136.89, 131.65, 129.81, 123.54 114.13, 113.51, 104.19, 104.10, 69.87, 69.52, 55.41, 31.73, 29.38, 29.34, 25.87, 25.85, 22.75, 14.16. **MS (MALDI-TOF):** *m/z* = 451.41 $[M]^+$ (100%). **UV-vis** (DCM): λ_{max} (nm) (ε ($dm^3 \cdot mol^{-1} \cdot cm^{-1}$)) = 356 ($1.76 \cdot 10^4$).

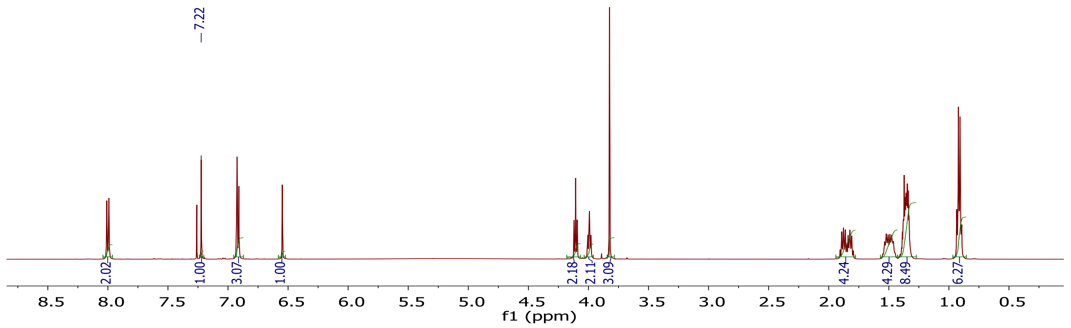

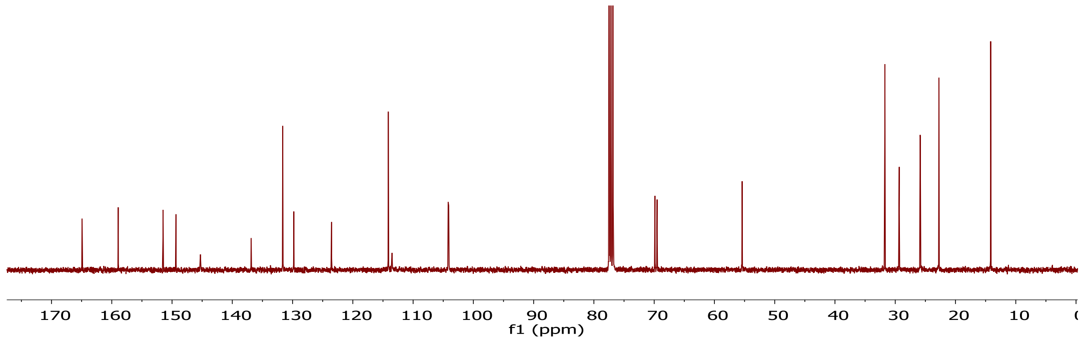

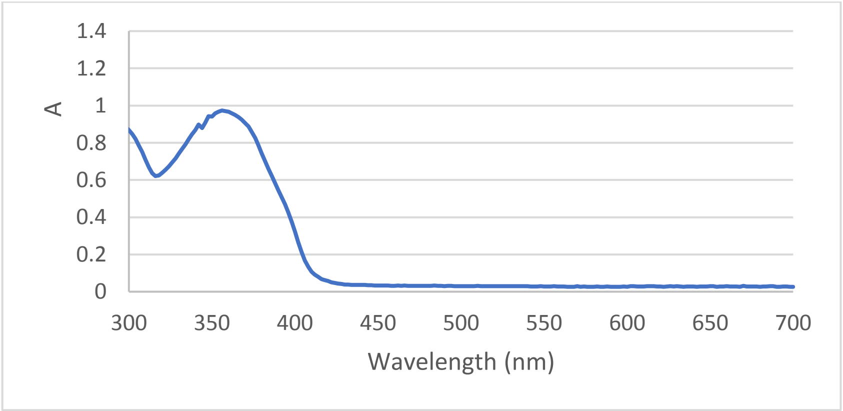

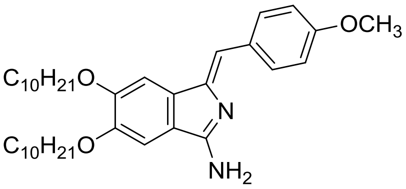

(*Z*)-5,6-bis(decyloxy)-1-[(4-methoxy)benzylidene]-1*H*-isoindol-3-amine (17c)

Synthesised from 2-bromo-4,5-bis(decyloxy)-benzamidine hydrochloride **16c** following the general aminoisoindolene synthesis giving **17c** as yellow needles (510 mg, 30%). **M.p.** 136.6 °C. **^{1}H NMR** (500 MHz, $CDCl_3$): δ = 7.95 (d, 2H, *J* = 8.8 Hz), 7.20 (s,1H), 7.03 (s,1H), 6.92 (d, 2H, *J* = 8.8 Hz), 6.55 (s, 1H), 4.10 (t, 2H, *J* = 6.6 Hz), 4.00 (t, 2H, *J* = 6.6 Hz), 3.82 (s, 3H, OCH_3), 1.95-1.75 (m, 4H), 1.58-1.41 (m, 4H), 1.39-1.27 (m, 24H), 0.96-0.87 (m, 6H). **^{13}C NMR** (100.6 MHz, $CDCl_3$): δ = 164.61, 159.09, 151.87, 149.54, 143.87, 136.40, 131.60, 129.39, 122.91, 114.22, 113.48, 104.41, 104.05, 69.87, 69.54, 55.41, 32.06, 29.78, 29.74, 29.59, 29.57, 29.50, 29.41, 29.37, 26.20, 22.83, 14.26. **MS (MALDI-TOF):** *m/z* = 563.24 $[M]^+$ (100%). **UV-vis** (DCM): λ_{max} (nm) (ε ($dm^3 \cdot mol^{-1} \cdot cm^{-1}$)) = 356 ($3.11 \cdot 10^4$).

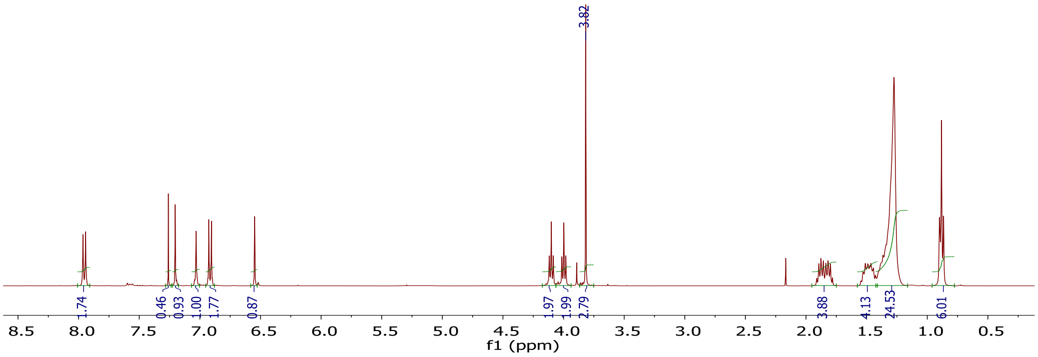

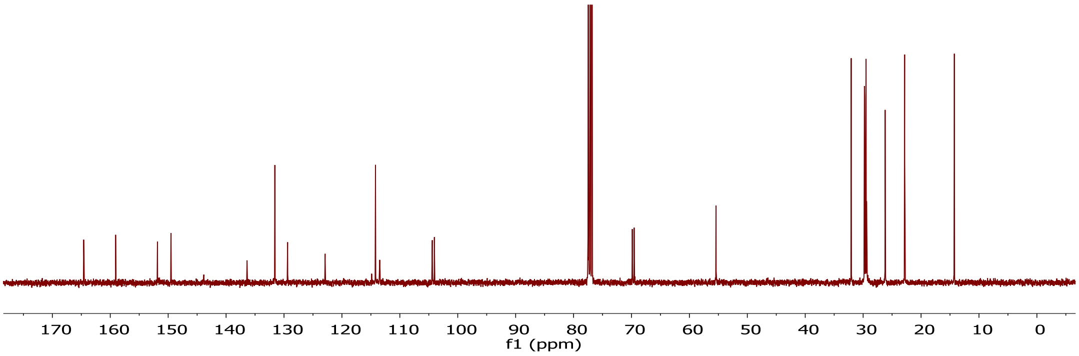

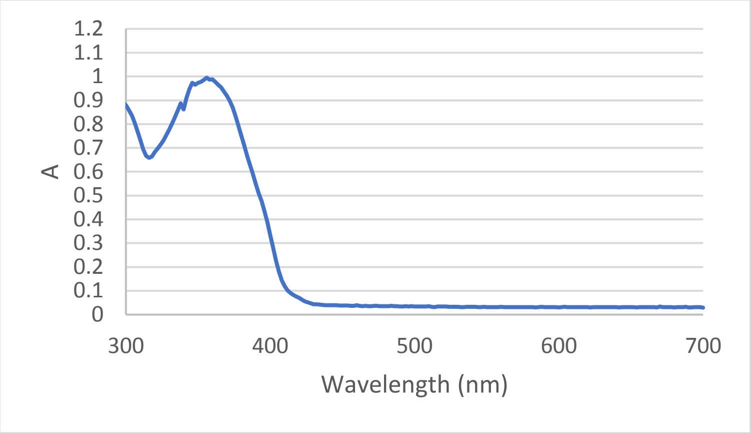

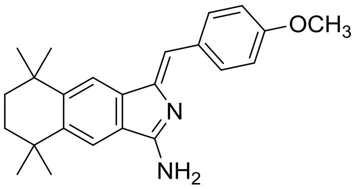

(*Z*)-1-[(4-methoxybenzylidene)]-5,5,8,8-tetramethyl-5H,6H,7H,8H-benzo[f]isoindol-3-amine (19)

Synthesised from 6-bromo-1,1,4,4-tetramethyl-1,2,3,4-tetrahydronaphthalene-7-imidamide hydrochloride **22** following the general aminoisoindolene synthesis giving **21** as yellow needles (400 mg, 36%). **M.p.** 202-205 °C. **^{1}H NMR** (500 MHz, $CDCl_3$): δ = 8.04 (d, *J* = 8.6 Hz, 2H), 7.70 (s, 1H), 7.41 (s, 1H), 6.93 (d, *J* = 8.6 Hz, 2H), 6.67 (s, 1H), 3.83 (s, 3H, OCH_3), 1.74 (s, 4H), 1.38 (s, 6H), 1.33 (s, 6H). **^{13}C NMR** (125.7 MHz, $CDCl_3$): δ = 164.73, 158.93, 147.05, 145.27, 144.74, 140.24, 131.74, 129.85, 128.65, 117.66, 117.13, 114.13, 113.76, 55.43, 35.19, 35.17, 35.13, 34.97, 32.38, 32.28. **MS (MALDI-TOF):** *m/z* = 362.20 $[M]^+$ (100%). **UV-vis** (DCM): λ_{max} (nm) (ε ($dm^3 \cdot mol^{-1} \cdot cm^{-1}$)) = 372 ($2.28 \cdot 10^4$).

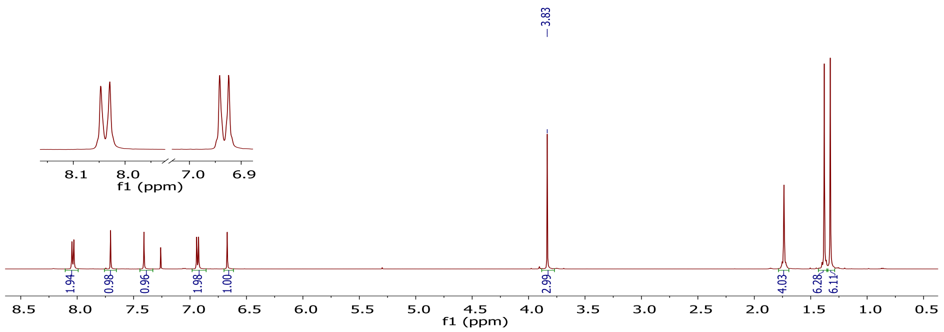

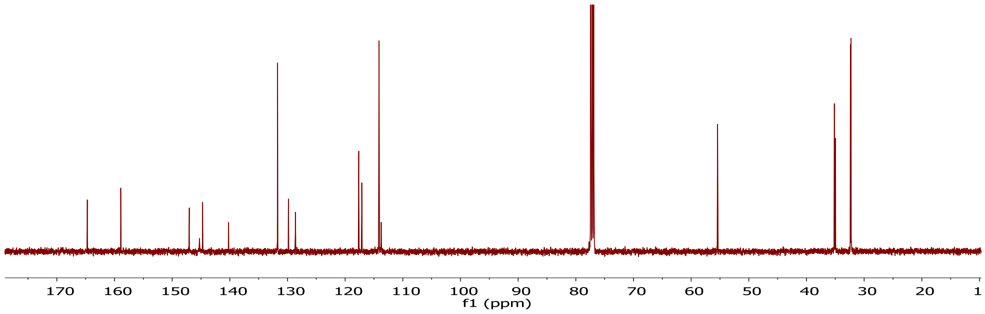

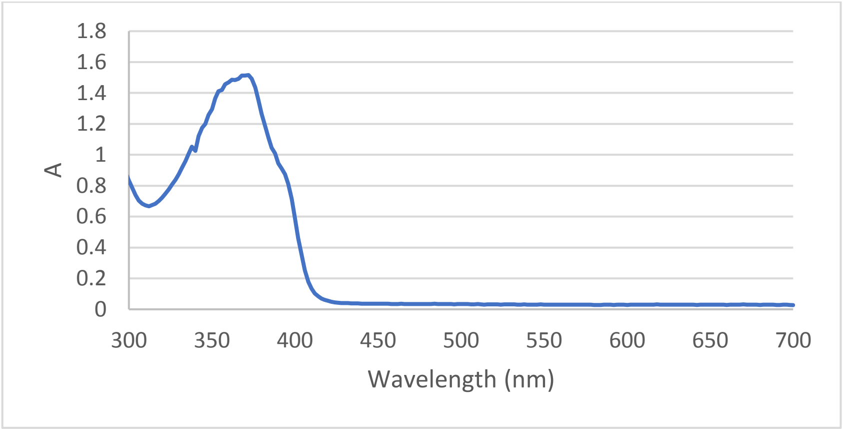

General Synthesis of TBTAP hybrids

Slow addition procedure:[14]

A suspension of phthalonitrile (1.2 mmol, 3eq) and $MgBr_2$ (110 mg, 0.6 mmol, 1.5eq) in dry diglyme (0.5 ml) was stirred for 10 min in an oil bath (220°C), under an argon atmosphere. A solution of aminoisoindolene (0.4 mmol, 1eq) and phthalonitrile (0.4 mmol, 1eq) in dry diglyme (1 ml) was added dropwise over 1 hour using a syringe pump. Finally, a third solution of DABCO (67.5 mg, 0.6 mmol, 1.5eq) and phthalonitrile (0.4 mmol, 1eq) in dry diglyme (0.5 ml) was added dropwise over 1 hour and heating continued for a further 0.5 h at 220°. The solvent was removed under an argon stream while cooling. A 1:1 mixture of DCM:MeOH (20 ml) was added and the mixture sonicated. The solvents were removed under vacuum and the crude mixture purified by two consecutive flash chromatography columns. First, using DCM→DCM:Et_3N (20:1)→DCM:THF:Et_3N (10:4:1) as eluent, the fractions were collected according to their colours. Only the green fraction was subjected to the second column using PE:THF:MeOH (10:3:1) as eluent. Recrystallisation from acetone and EtOH gave the pure compounds.

One-pot procedure:

A suspension of phthalonitrile (2 mmol, 5 eq), $MgBr_2$ (110 mg, 0.6 mmol, 1.5 eq), aminoisoindolene (0.4 mmol, 1 eq) and DABCO (67.5 mg, 0.6 mmol, 1.5 eq) in dry diglyme (2 ml) was stirred for 1.5 h in an oil bath at 220°C, under an argon atmosphere. Then, the solvent was removed under an argon stream while cooling and the residue worked up and separated as previously described.

"Opposite" Series

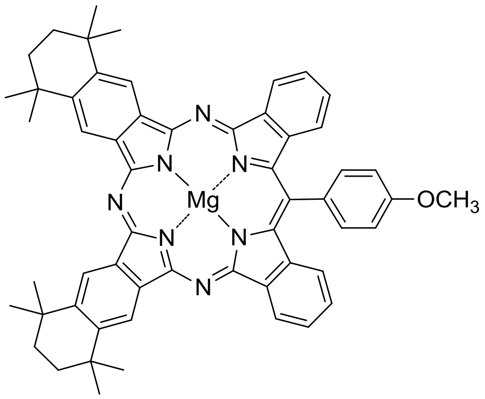

ABBA-TBTAP 8

Following the general syntheses of TBTAPs using 6,7-Dicyano-1,2,3,4-tetrahydro-1,1,4,4-tetramethylnaphthalene **4** and aminoisoindolene **6**. This compound is isolated as the major product, [slow addition (53 mg, 31%), one-pot method (39 mg, 22%)]. **Chemical Formula:** $C_{56}H_{51}MgN_7O$. **M.p.** > 300°C. **^{1}H NMR** (500 MHz, THF-d_8): δ = 9.57 (d, J = 7.4 Hz, 2H), 9.50 (s, 2H), 9.49 (s, 2H), 8.02 (d, J = 8.4 Hz, 2H), 7.87 (t, J = 7.2 Hz, 2H), 7.58 (t, J = 7.2 Hz, 2H), 7.51 (d, J = 8.4 Hz, 2H), 7.21 (d, J = 8.1 Hz, 2H), 4.20 (s, 3H, OCH_3), 2.10 (s, 8H, CH_2), 1.82 (s, 24H). **^{13}C NMR** (125.7 MHz, THF-d_8): δ = 161.91, 157.78, 154.02, 152.17, 147.87, 147.49, 142.08, 141.13, 139.84, 139.19, 138.89, 136.19, 134.12, 127.89, 127.06, 126.82, 125.63, 123.62, 121.91, 121.65, 115.20, 56.12, 36.68, 36.63, 36.59, 33.32. **MS (MALDI-TOF):** m/z = 861.31 $[M]^+$ (100%). **UV-vis** (THF): λ_{max} (nm) (ε ($dm^3 \cdot mol^{-1} \cdot cm^{-1}$)) = 676 ($1.88 \cdot 10^5$), 652 ($1.14 \cdot 10^5$), 598 ($3.08 \cdot 10^4$), 444 ($2.67 \cdot 10^4$), 398 ($6.44 \cdot 10^4$).

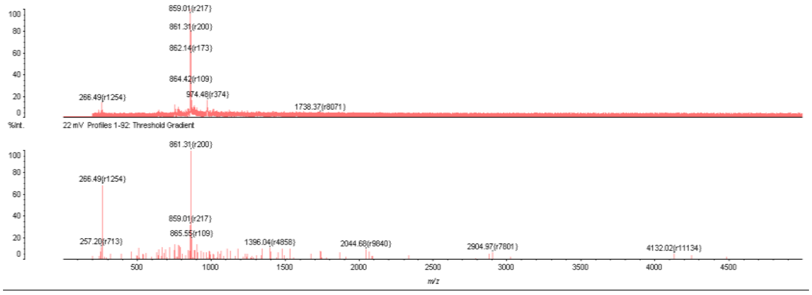
859.01(r217)
861.31(r200)
862.14(r173)
864.42(r109)
974.48(r374)
266.49(r1254)
1738.37(r8071)
%Int.
22 mV Profiles 1-92: Threshold Gradient
861.31(r200)
266.49(r1254)
859.01(r217)
865.55(r109)
257.20(r713)
1396.04(r4858)
2044.68(r9840)
2904.97(r7801)
4132.02(r11134)
m/z

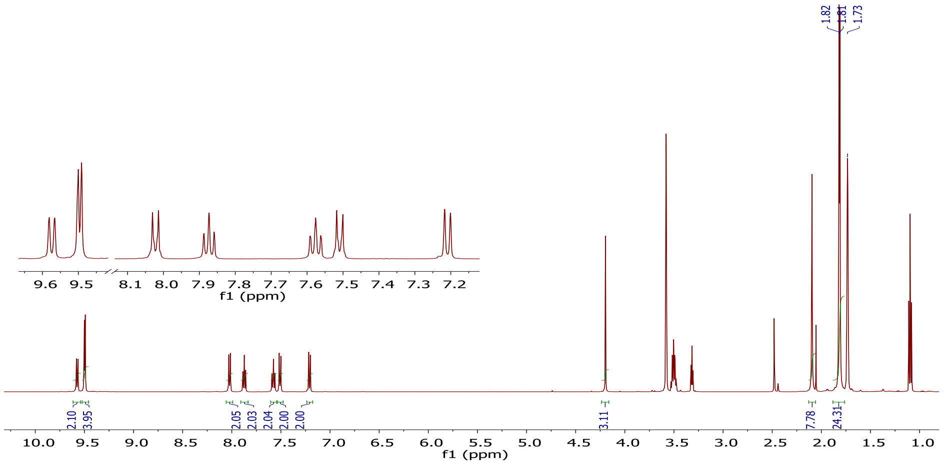
1.82
1.81
1.73
f1 (ppm)
2.10
3.95
2.05
2.03
2.04
2.00
2.00
3.11
7.78
24.31
f1 (ppm)

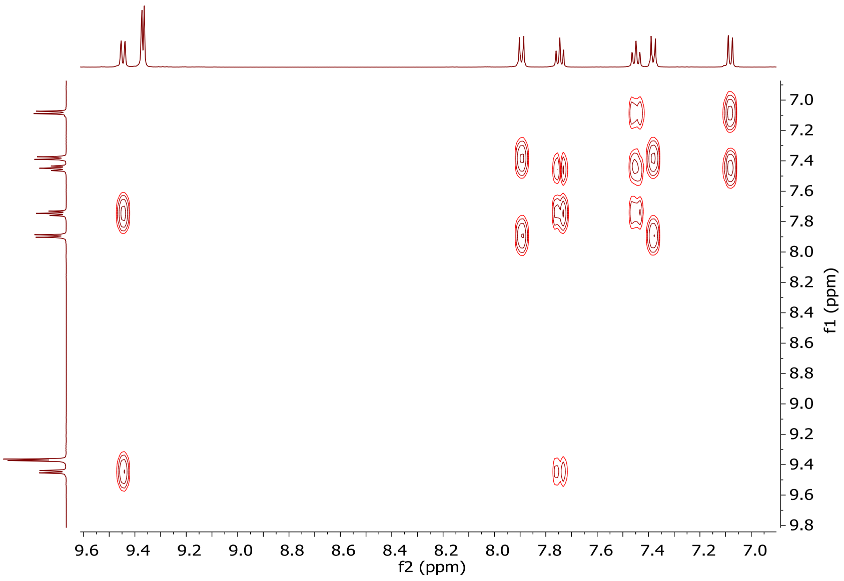
f1 (ppm)
f2 (ppm)

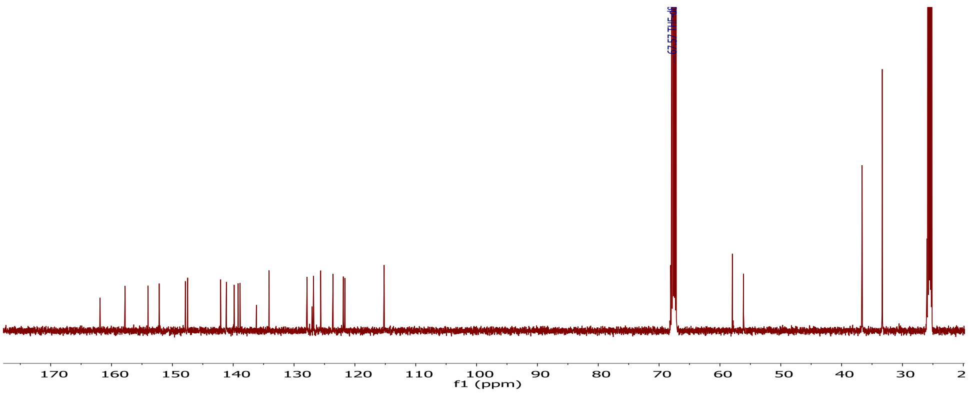
f1 (ppm)

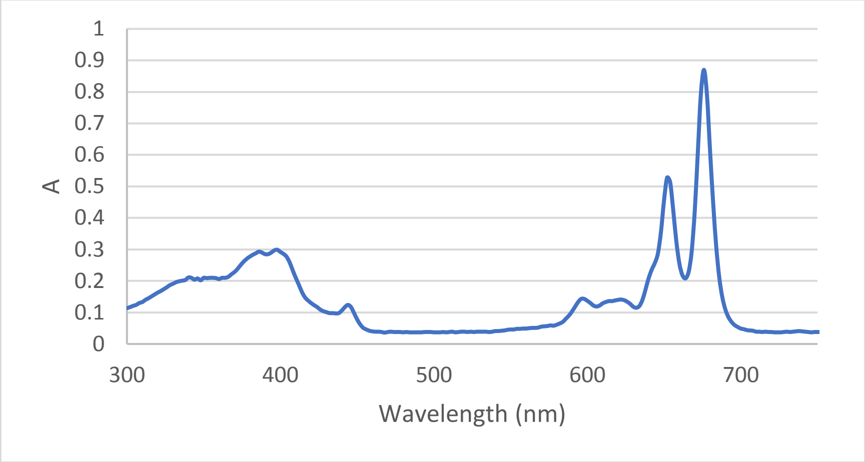

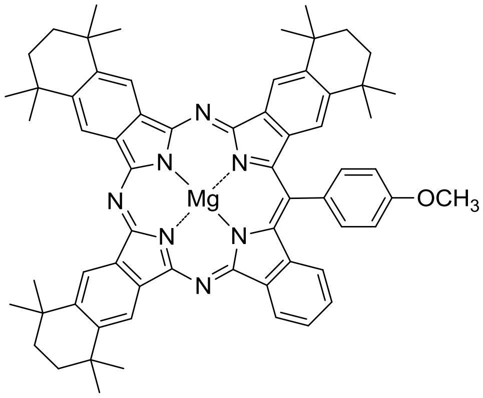

ABBB-TBTAP 7

Following the slow addition general synthesis of TBTAPs using 6,7-Dicyano-1,2,3,4-tetrahydro-1,1,4,4-tetramethylnaphthalene **4** and aminoisoindolene **6**, this compound is isolated alongside ABBA-TBTAP **8** (30 mg, 8%). **Chemical Formula:** $C_{64}H_{65}MgN_7O$. **M.p.** > 300°C. **^{1}H NMR** (500 MHz, THF-d_8): = δ 9.58 (d, *J* = 7.5 Hz, 1H), 9.57-9.49 (m, 5H), 8.04 (d, *J* = 8.3 Hz, 2H), 7.87 (t, *J* = 7.2 Hz, 1H), 7.59-7.54 (m, 3H), 7.38 (d, *J* = 8.0 Hz, 1H), 7.17 (s, 1H), 4.20 (s, 3H, OCH_3), 2.09 (s, 12H), 1.82 (s, 24H), 1.36 (s, 12H). **^{13}C NMR** (125.7 MHz, THF-d_8): δ = 161.88, 153.96, 153.27, 152.94, 147.56, 147.25, 147.17, 145.14, 144.85, 142.96, 142.02, 140.96, 139.91, 139.36, 139.10, 138.89, 138.20, 136.50, 134.33, 127.65, 126.74, 125.67, 124.53, 123.58, 121.80, 121.73, 121.58, 120.80, 115.48, 56.22, 36.67, 36.60, 36.31, 36.11, 33.34, 33.19, 33.17. **MS (MALDI-TOF):** *m/z* = 971.92 $[M]^+$ (100%). **UV-vis** (THF): λ_{max} (nm) (ε ($dm^3 \cdot mol^{-1} \cdot cm^{-1}$)) = 682 ($5.89 \cdot 10^4$), 658 ($3.49 \cdot 10^4$), 602 ($9.75 \cdot 10^3$), 448 ($8.22 \cdot 10^3$), 398 ($2.21 \cdot 10^4$).

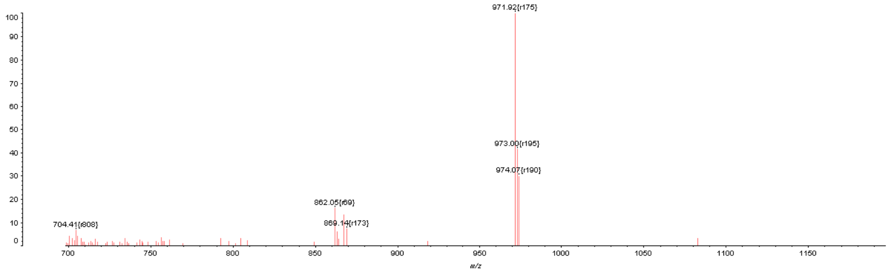
971.92[r175]
973.00[r195]
974.07[r190]
862.05[r69]
869.14[r173]
704.41[r808]
m/z

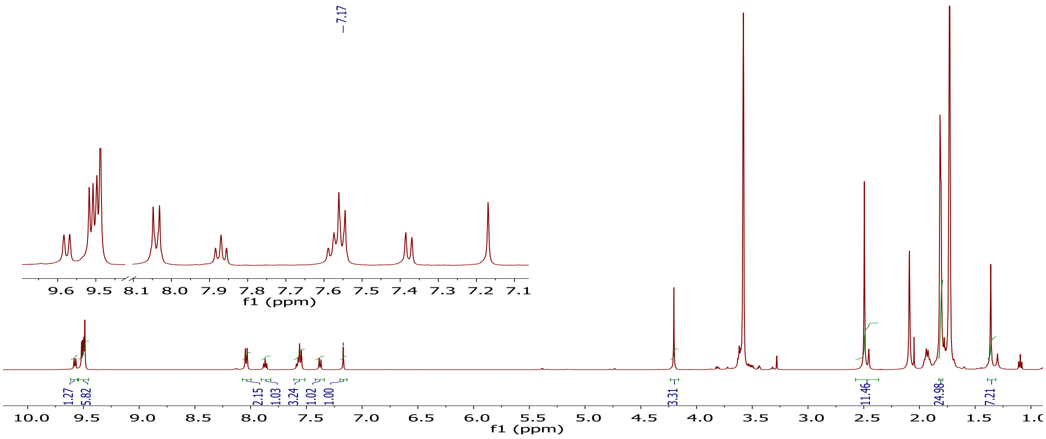
7.17
f1 (ppm)
1.27
5.82
2.15
1.03
3.24
1.02
1.00
3.31
11.46
24.98
7.21
f1 (ppm)

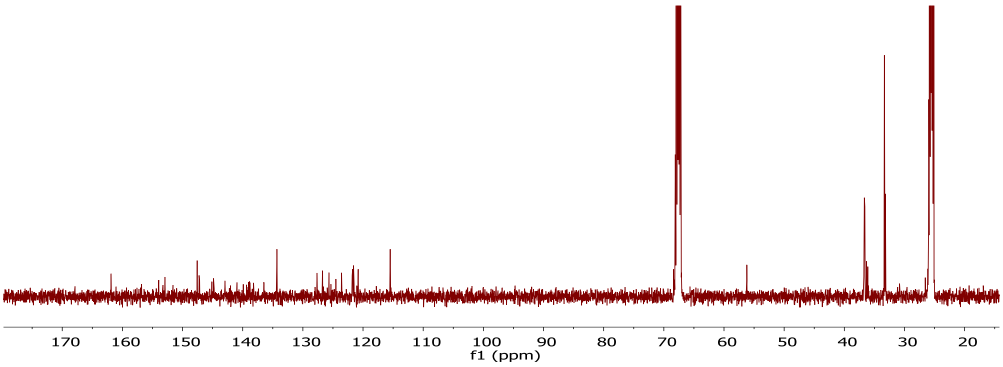
f1 (ppm)

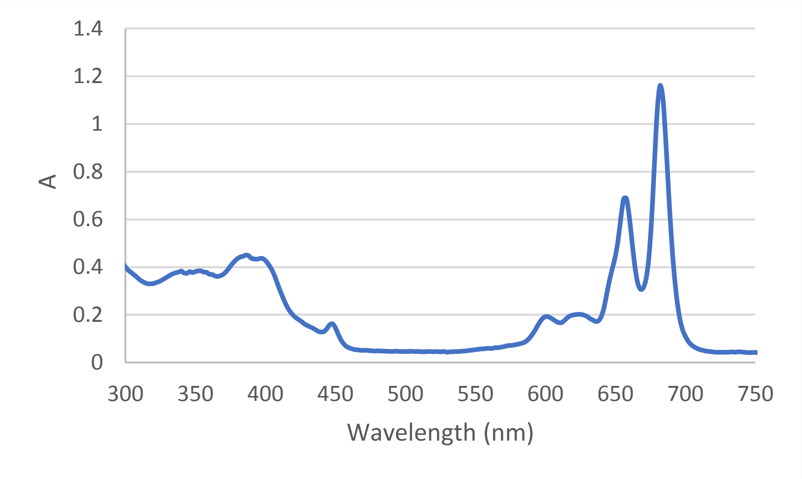
A
Wavelength (nm)

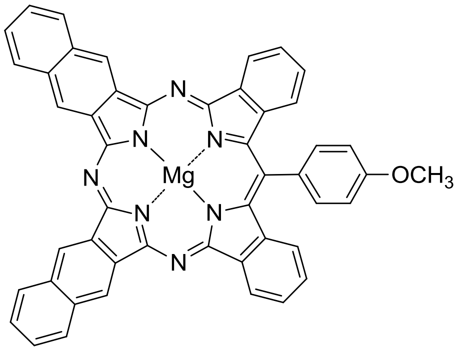

ABBA-TBTAP 10

Synthesised following the general synthesis of TBTAPs using 2,3-Naphthalonitrile **9** and aminoisoindolene **6** by the slow addition method. Recrystallisation from acetone and ethanol gave the title compound as purple crystals (11.1 mg, 8%). **Chemical Formula:** $C_{48}H_{27}MgN_7O$. **M.p.** > 300°C. **^{1}H NMR** (500 MHz, THF-d_8): δ = 9.99 (s, 2H), 9.91 (s, 2H), 9.54 (d, *J* = 7.5 Hz, 2H), 8.64 (ddd, *J* = 14.5, 6.1, 3.2 Hz, 4H), 8.01 (d, *J* = 8.4 Hz, 2H), 7.86 (m, 6H), 7.56 (ddd, *J* = 8.0, 6.8, 1.2 Hz, 2H), 7.51 (d, *J* = 8.4 Hz, 2H), 7.16 (d, *J* = 8.0 Hz, 2H), 4.19 (s, 3H, OCH_3). **^{13}C NMR** (125.7 MHz, THF-d_8): δ = 161.91, 158.71, 154.28, 152.29, 141.62, 141.00, 139.49, 138.56, 138.50, 136.04, 135.60, 135.33, 134.01, 130.93, 130.84, 128.04, 127.91, 127.89, 127.68, 126.75, 125.56, 123.54, 123.44, 123.06, 115.29, 56.12. **MS (MALDI-TOF):** *m/z* = 740.35 $[M]^+$ (100%). **UV-vis** (THF): λ_{max} (nm) (ε ($dm^3 \cdot mol^{-1} \cdot cm^{-1}$)) = 709 ($8.02 \cdot 10^4$), 681 ($5.39 \cdot 10^4$), 621 ($1.36 \cdot 10^4$), 404 ($3.84 \cdot 10^4$).

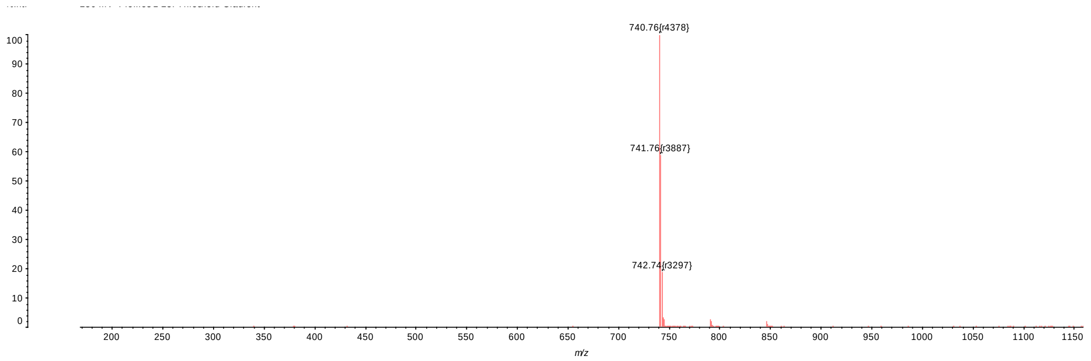

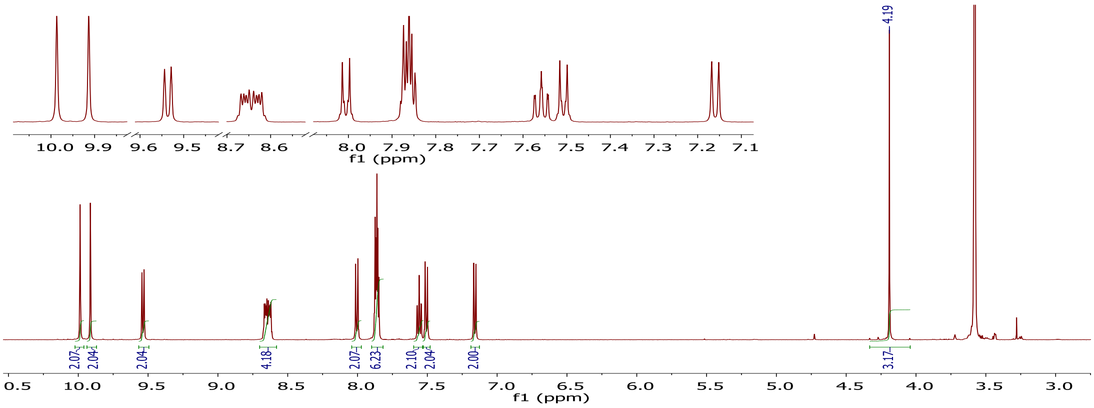

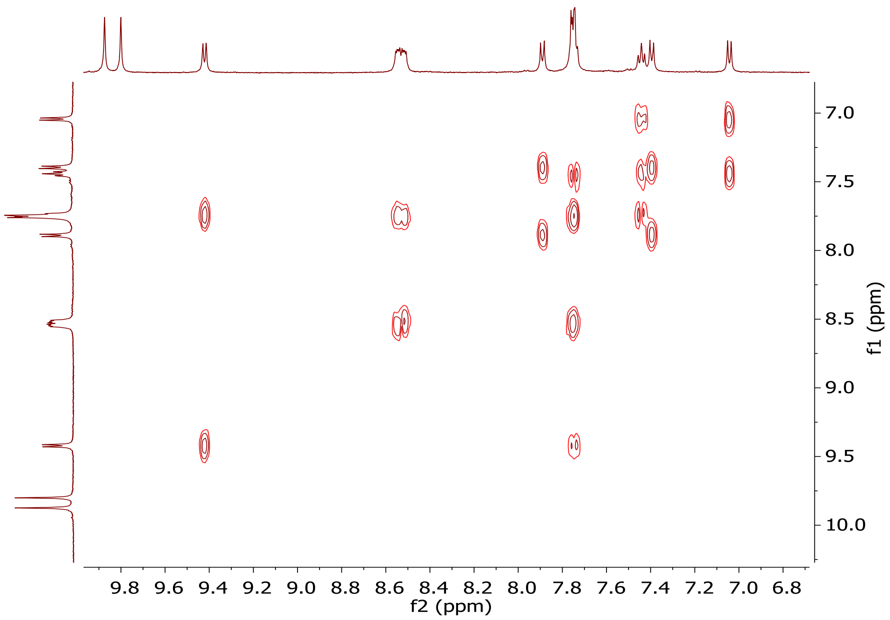
9.8 9.6 9.4 9.2 9.0 8.8 8.6 8.4 8.2 8.0 7.8 7.6 7.4 7.2 7.0 6.8
f2 (ppm)
7.0
7.5
8.0
8.5
9.0
9.5
10.0
f1 (ppm)

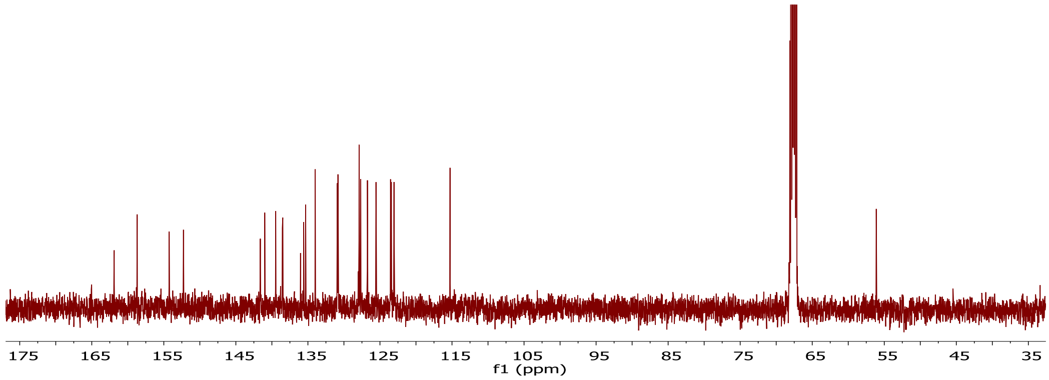
175 165 155 145 135 125 115 105 95 85 75 65 55 45 35
f1 (ppm)

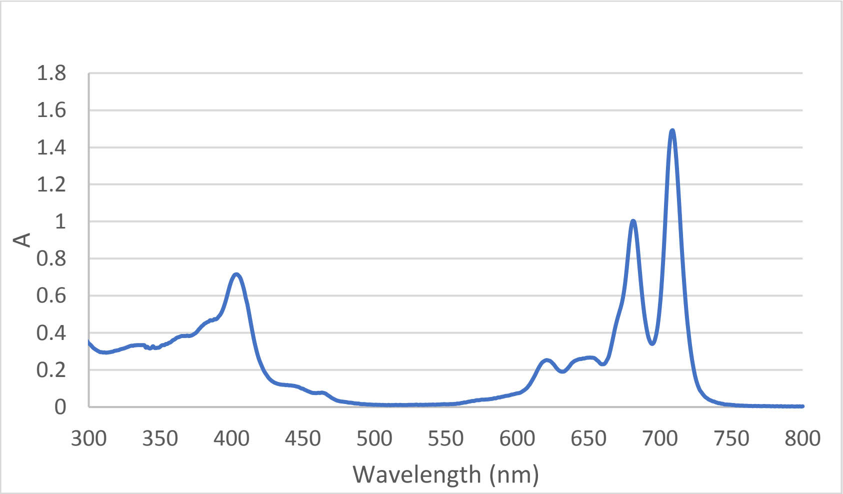
1.8
1.6
1.4
1.2
1
0.8
0.6
0.4
0.2
0
A
300 350 400 450 500 550 600 650 700 750 800
Wavelength (nm)

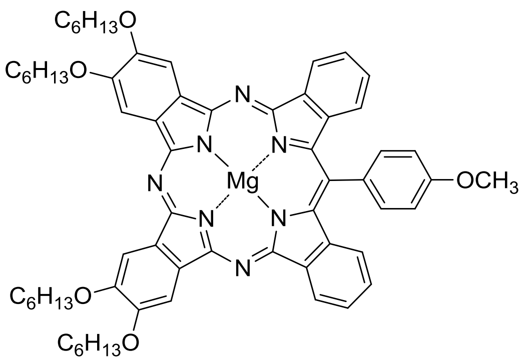

ABBA-TBTAP 14b

Synthesised following the general synthesis of TBTAPs using 1,2-dicyano-4,5-bis(hexyloxy)benzene **12b** and aminoisoindolene **6** by the one-pot procedure. After chromatography, careful recrystallization from acetone and ethanol gave an analytically pure sample (6.6 mg, 4%). **Chemical Formula:** $C_{64}H_{71}MgN_7O_5$. **M.p.** 222°C. **^{1}H NMR** (400 MHz, THF-d_8): δ = 9.56 (d, *J* = 7.4 Hz, 2H), 8.97 (s, 2H), 8.95 (s, 2H), 8.02 (d, *J* = 8.5 Hz, 2H), 7.87 (t, *J* = 7.4 Hz, 2H), 7.58 (ddd, *J* = 8.1, 6.9, 1.2 Hz, 2H), 7.50 (d, *J* = 8.5 Hz, 2H), 7.22 (d, *J* = 8.1 Hz, 2H), 4.62-4.57 (m, 8H), 4.19 (s, 3H), 2.17-2.05 (m, 8H), 1.84-1.76 (m, 8H), 1.62-1.47 (m,16H), 1.11-1.62 (m, 12H). (^{13}C NMR could not be obtained due to low solubility/aggregation). **MS (MALDI-TOF):** *m/z* = 1042.26 $[M]^+$ (100%). **UV-vis** (THF): λ_{max} (nm) (ε ($dm^3 \cdot mol^{-1} \cdot cm^{-1}$)) = 670 ($7.55 \cdot 10^4$), 648 ($5.31 \cdot 10^4$), 594 ($2.14 \cdot 10^4$), 416 ($1.17 \cdot 10^5$).

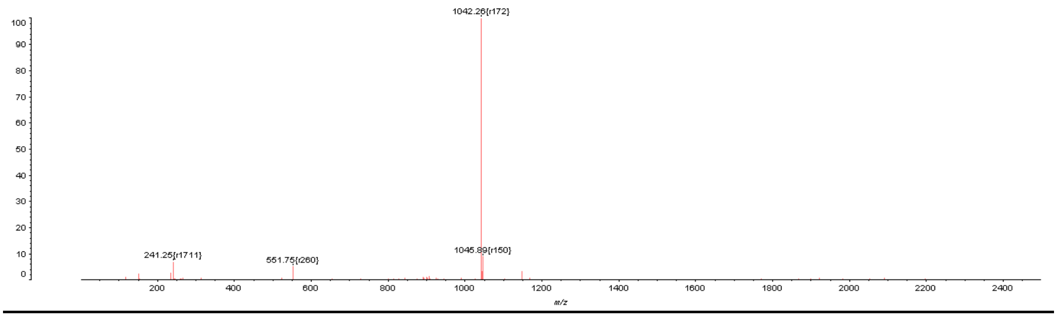

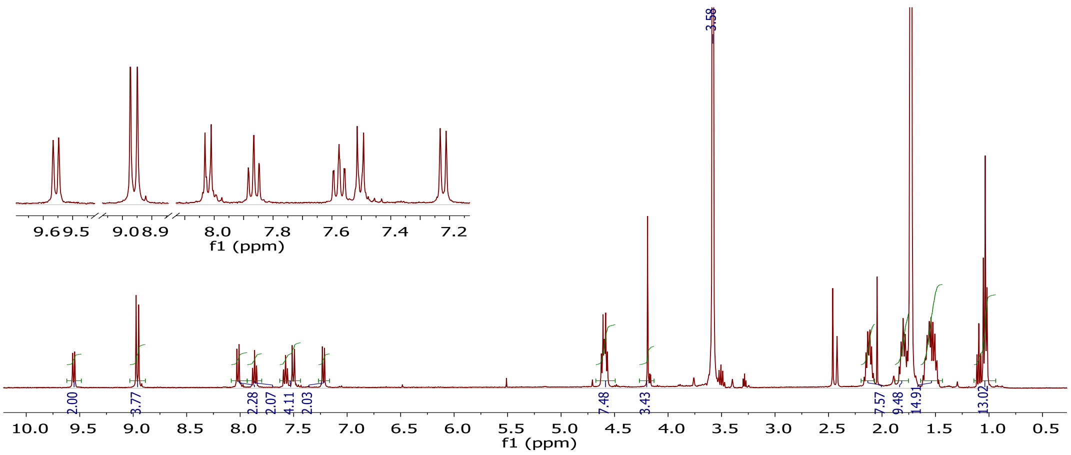

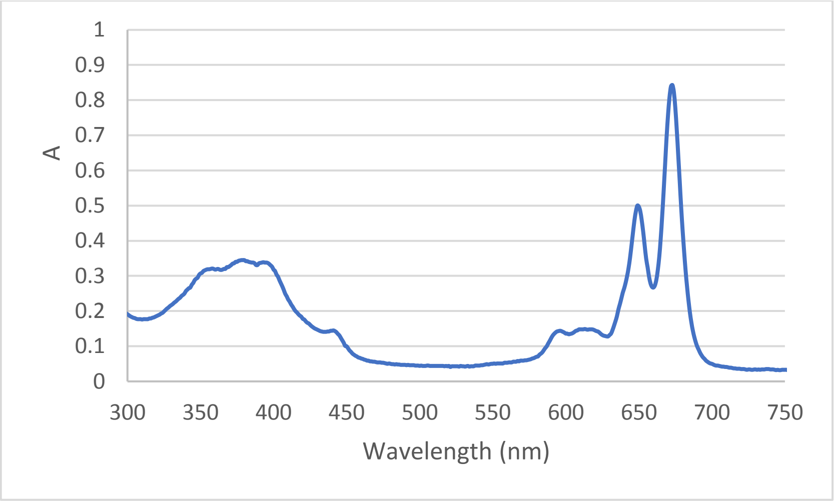

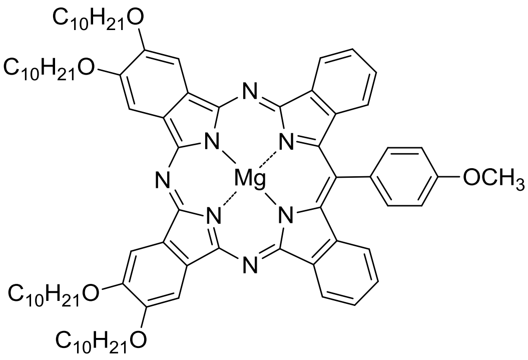

ABBA-TBTAP 14c

Synthesised following the general synthesis of TBTAPs using 1,2- dicyano-4,5- bis(decyloxy)benzene **12c** and aminoisoindolene **6** and the one-pot procedure. After chromatography, careful recrystallisation from acetone and ethanol gave and analytically pure sample of the title compound as green crystals (73 mg, 28%). **Chemical Formula:** $C_{80}H_{103}MgN_7O_5$. **M.p.** 173°C. **^{1}H NMR** (500 MHz, THF-d_8): δ = 9.68 (d, *J* = 7.5 Hz, 2H), 9.09 (s, 2H), 9.06 (s, 2H), 8.14 (d, *J* = 8.4 Hz, 2H), 7.98 (t, *J* = 7.2 Hz, 2H), 7.69 (t, *J* = 7.9 Hz, 2H), 7.62 (d, *J* = 8.4 Hz, 2H), 7.34 (d, *J* = 8.1 Hz, 2H), 4.73-4.68 (m, 8H), 4.31 (s, 3H, OCH_3), 2.26-2.21 (m, 8H), 1.92-1.89 (m, 8H), 1.68-1.46 (m, 48H), 1.06-1.03 (m, 12H). (^{13}C NMR could not be obtained due to low solubility/aggregation). **MS (MALDI-TOF):** *m/z* = 1267.31[M]$^+$ (100%). **UV-vis** (THF): λ_{max} (nm) (ε (dm^3·mol^{-1}·cm^{-1})) = 673 (4.13·10^5), 649 (2.45·10^5), 596 (7.06·10^4).

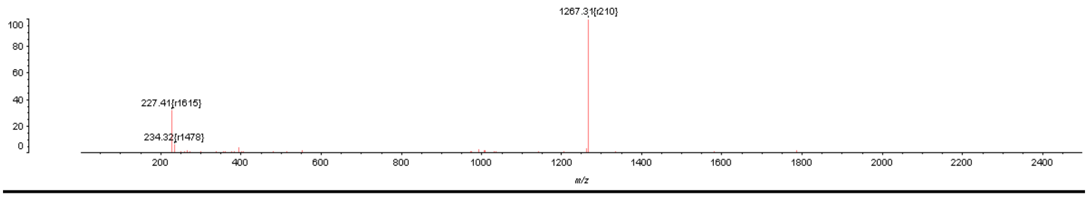

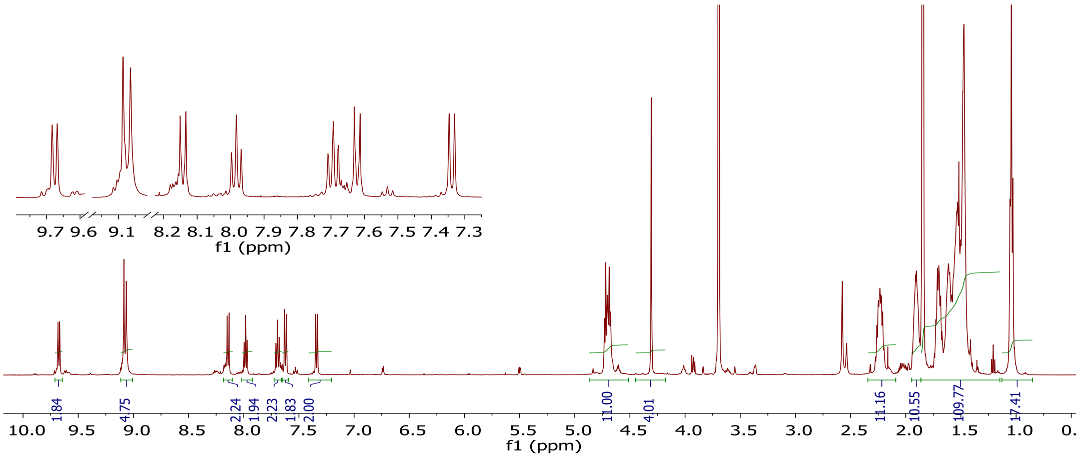

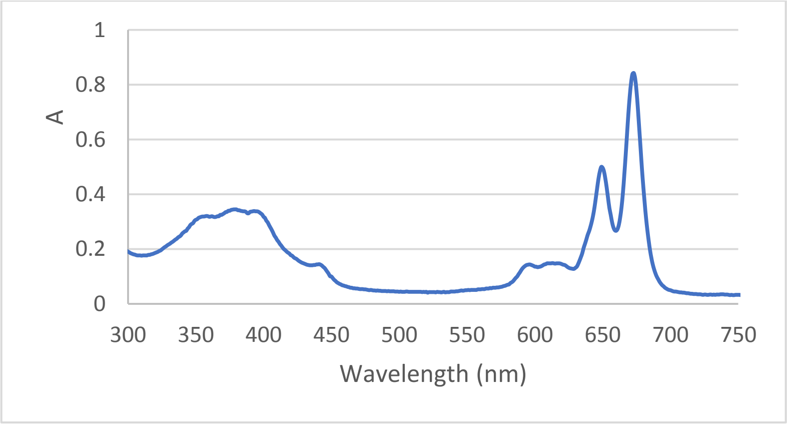

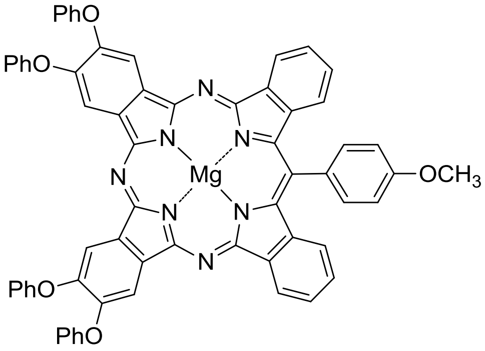

ABBA-TBTAP 14d

Synthesised following the general synthesis of TBTAPs using 1,2-Dicyano-4,5-bis(phenoxy)benzene **12d** and aminoisoindolene **6** and the one-pot procedure. Recrystallization from acetone and ethanol gave the title compound as a green powder (105 mg, 52%). **Chemical Formula:** $C_{64}H_{39}MgN_7O_5$. **M.p.** > 300°C. **^{1}H NMR** (500 MHz, THF-d_8): δ = 9.47 (d, *J* = 7.6 Hz, 2H), 9.14 (s, 2H), 9.11 (s, 2H), 8.02 (d, *J* = 8.6 Hz, 2H), 7.86 (t, *J* = 7.3 Hz, 2H), 7.59 (t, *J* = 7.4 Hz, 2H), 7.51(d, *J* = 8.6 Hz, 2H), 7.47-7.40 (m, 6H), 7.30-7.15 (m, 16H), 4.18 (s, 3H, OCH_3). **^{13}C NMR** (100.6 MHz, THF-d_8): δ = 162.03, 159.40, 159.16, 155.32, 152.72, 152.56, 151.30, 150.54, 144.05, 141.30, 140.18, 137.65, 137.09, 135.84, 134.14, 130.82, 130.73, 128.37, 127.56, 126.70, 125.92, 124.27, 124.04, 123.66, 119.22,

118.80, 116.36, 115.61, 115.33, 56.15. **MS (MALDI-TOF):** *m/z* = 1009.38 $[M]^+$ (100%). **UV-vis** (THF): λ_{max} (nm) (ε ($dm^3 \cdot mol^{-1} \cdot cm^{-1}$)) = 675 ($1.45 \cdot 10^5$), 652 ($9.12 \cdot 10^4$), 597 ($2.65 \cdot 10^4$), 446 ($2.95 \cdot 10^4$), 399 ($5.61 \cdot 10^4$).

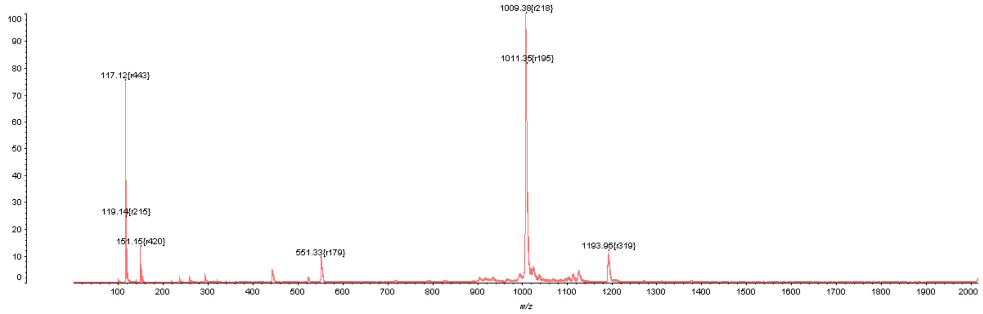

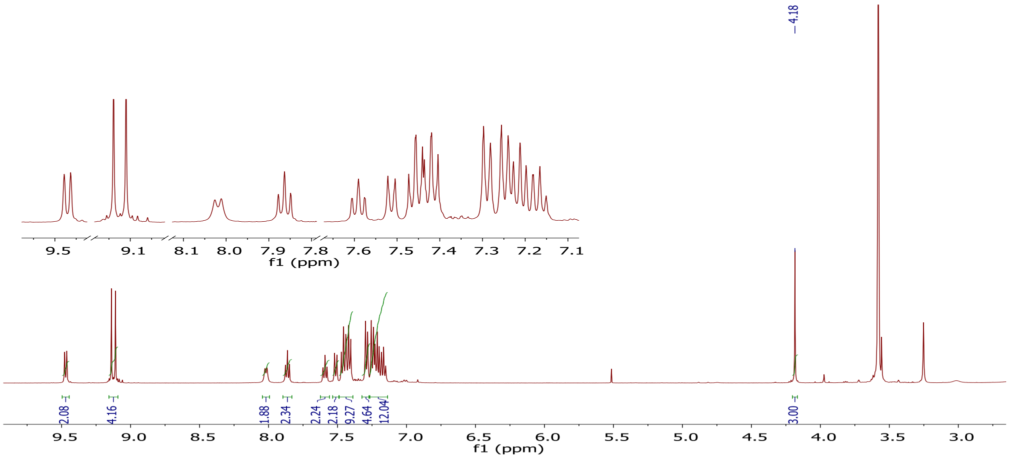

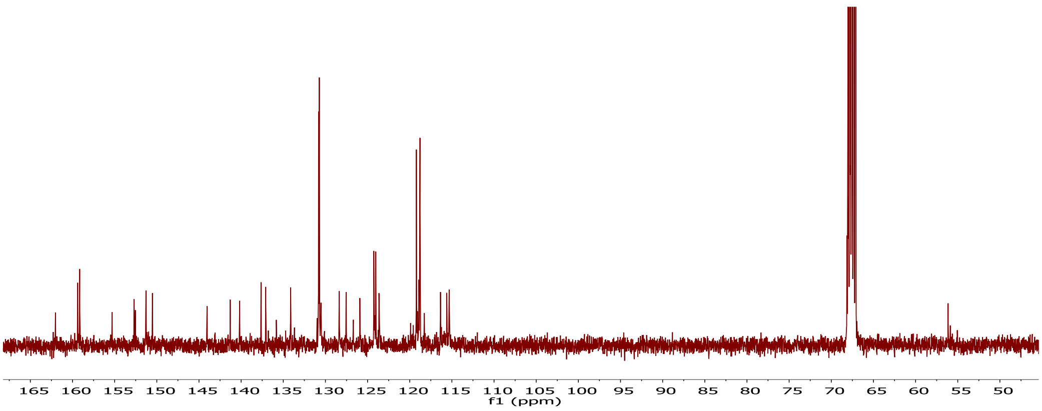

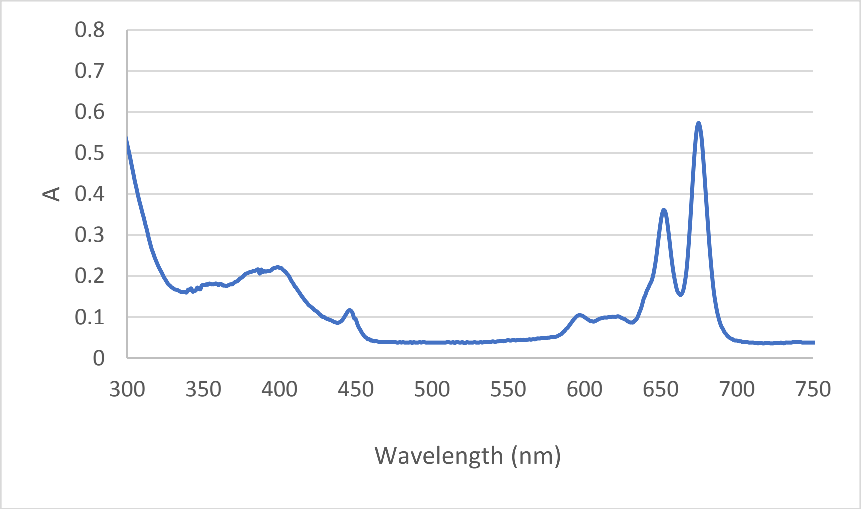

"Adjacent" series

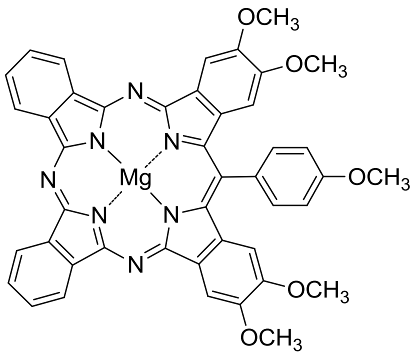

ABBA-TBTAP 18a

Synthesised following the general synthesis of TBTAPs using phthalonitrile and aminoisoindolene **17a** in the one-pot procedure. After chromatography, careful recrystallization from acetone and ethanol gave an analytically pure sample of the title compound as green crystals, (9.2 mg, 6%). **Chemical Formula:** $C_{44}H_{31}MgN_7O_5$. **M.p.** > 300°C. **^{1}H NMR** (500 MHz, THF-d_8): δ = 9.55-9.52 (m, 4H), 9.06 (s, 2H), 8.17-8.18 (m, 4H), 8.10 (d, J = 8.1 Hz, 2H), 7.61 (d, J = 8.1 Hz, 2H), 6.84 (s, 2H), 4.31 (s, 6H), 4.14 (s, 3H), 3.77 (s, 6H). **^{13}C NMR** (125.7 MHz, THF-d_8): δ = 161.92,

155.24, 152.78, 152.56, 151.39, 151.26, 143.77, 140.89, 140.76, 136.22, 134.82, 134.32, 134.19, 129.48, 129.32, 125.00, 123.66, 123.45, 115.62, 107.83, 104.91, 56.54, 56.24, 55.86. **MS (MALDI-TOF):** *m/z* = 761.82 $[M]^+$ (100%). **UV-vis** (THF): λ_{max} (nm) (ε ($dm^3 \cdot mol^{-1} \cdot cm^{-1}$)) = 673 ($1.64 \cdot 10^5$), 650 ($9.82 \cdot 10^4$), 596 ($2.84 \cdot 10^4$), 446 ($3.10 \cdot 10^4$), 395 ($9.29 \cdot 10^4$).

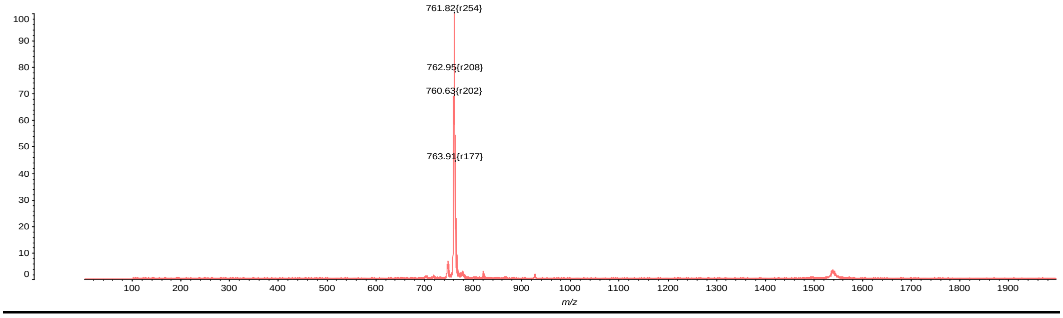

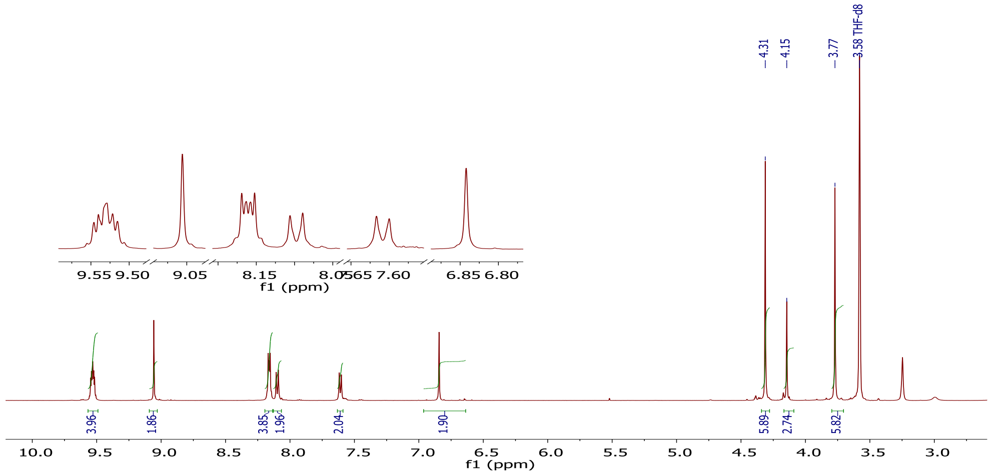

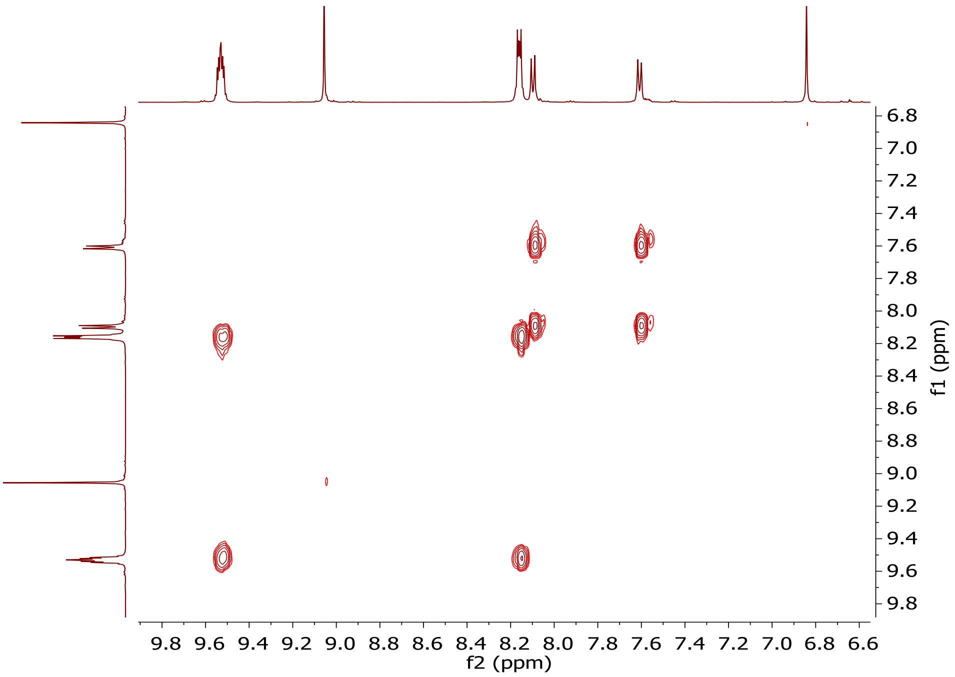

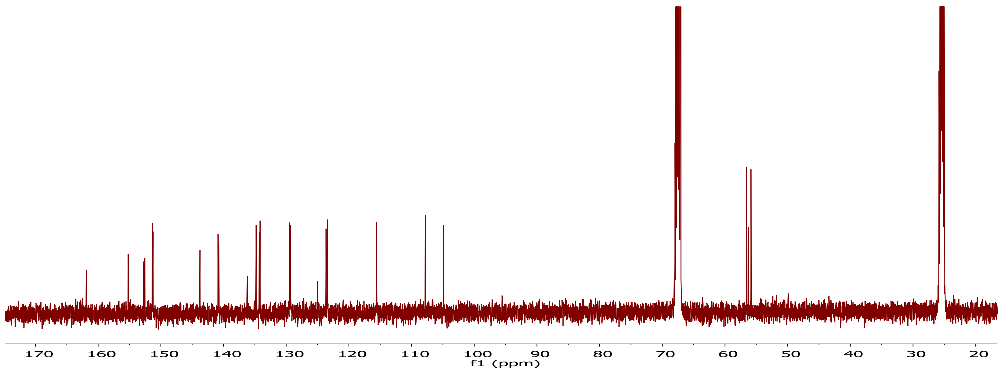

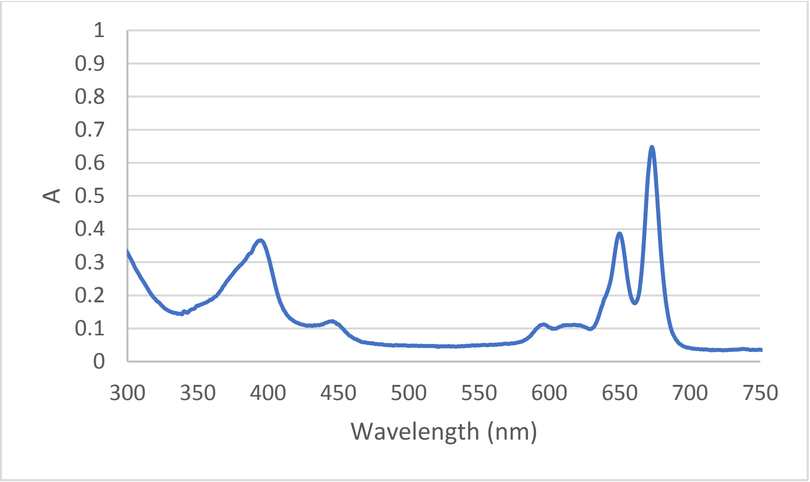

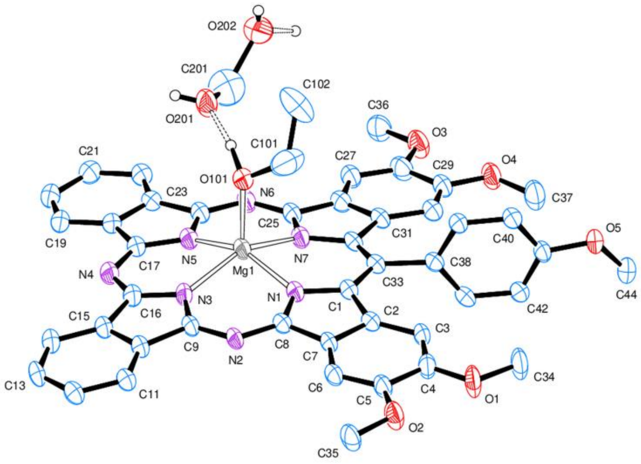

(further crystallography details below)

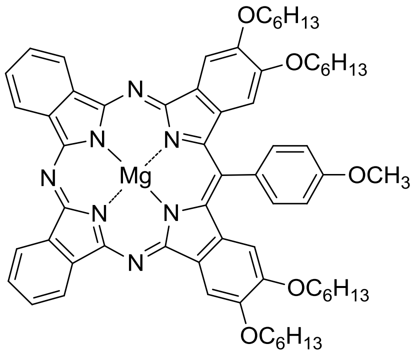

ABBA-TBTAP 18b

Synthesised following the general synthesis of TBTAPs using phthalonitrile and aminoisoindolene **17b** in the one-pot procedure. After chromatography, careful recrystallisation from acetone and ethanol gave an analytically pure sample of the title compound as green crystals, (62 mg, 30%). **Chemical Formula:** $C_{64}H_{71}MgN_7O_5$. **M.p.** 159.2°C. **^{1}H NMR** (500 MHz, THF-d_8): δ = 9.54-9.51 (m, 4H), 9.04 (s, 2H), 8.16-8.14 (m, 4H), 8.08 (d, *J* = 8.5 Hz, 2H), 7.60 (d, *J* = 8.5 Hz, 2H), 6.84 (s, 2H), 4.54 (t, *J* = 6.5 Hz, 4H), 4.18 (s, 3H), 3.91 (t, *J* = 6.5 Hz, 4H), 2.11-2.01 (m, 4H), 1.96-1.86 (m, 4H,), 1.67-1.63 (m, 4H), 1.54-1.56 (m, 4H), 1.53-1.45 (m, 16H), 1.05-1.00 (m, 12H). (^{13}C NMR could not be obtained due to low solubility/aggregation). **MS (MALDI-TOF):** *m/z* = 1041.87 [M]$^+$ (100%). **UV-vis** (THF): λ_{max} (nm) (ε (dm^3·mol^{-1}·cm^{-1})) = 675 (1.00·10^5), 652 (5.82·10^4), 597 (1.53·10^4), 446 (1.57·10^4), 396 (5.15·10^4).

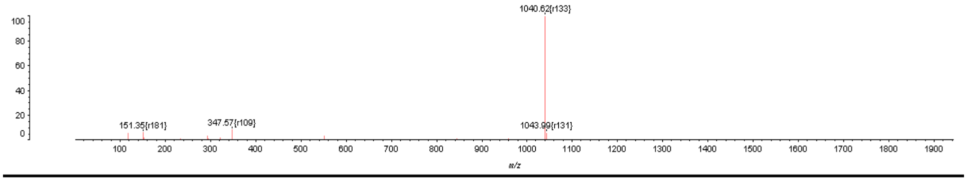

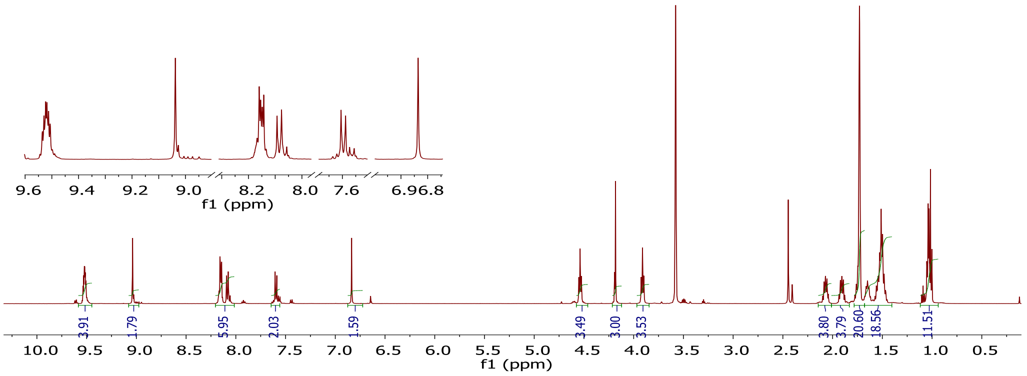

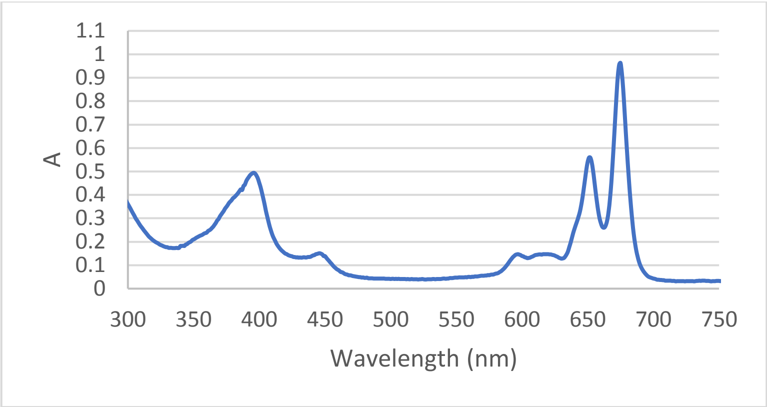

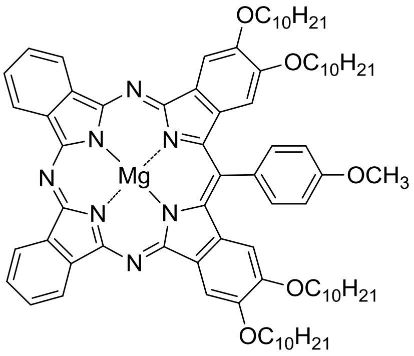

ABBA-TBTAP 18c

Following the general synthesis of TBTAPs using phthalonitrile and aminoisoindolene **17c** and one-pot reaction method. Recrystallization from acetone and ethanol gave the title compound as green crystals (9.3 mg, 4%). **Chemical Formula:** $C_{80}H_{103}MgN_7O_5$. **M.p.** 189.8°C. **^{1}H NMR** (500 MHz, THF-d_8): δ = 9.57-9.45 (m, 4H), 9.03 (s, 2H), 8.19-8.11 (m, 4H), 8.08 (d, J = 8.5 Hz, 2H), 7.60 (d, J = 8.5 Hz, 2H), 6.83 (s, 2H), 4.53 (t, J = 6.5 Hz, 4H), 4.18 (s, 3H, OCH_3), 3.91 (t, J = 6.5 Hz, 4H), 2.12-2.01 (m, 4H), 1.93-1.87 (m, 4H), 1.68-1.19 (m, 56H), 0.95-0.90 (m, 12H). (^{13}C NMR could not be obtained due to low solubility/aggregation). **MS (MALDI-TOF):** *m/z* = 1266.55 $[M]^+$ (100%). **UV-vis** (THF): λ_{max} (nm) (ε ($dm^3 \cdot mol^{-1} \cdot cm^{-1}$)) = 675 ($2.64 \cdot 10^5$), 652 ($1.56 \cdot 10^5$), 597 ($4.47 \cdot 10^4$), 447 ($4.73 \cdot 10^4$), 396 ($1.46 \cdot 10^5$).

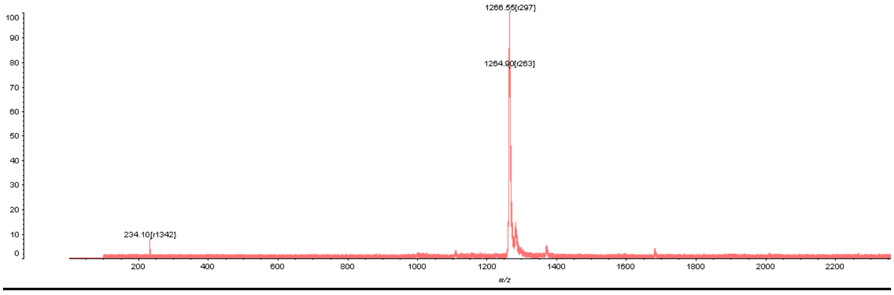

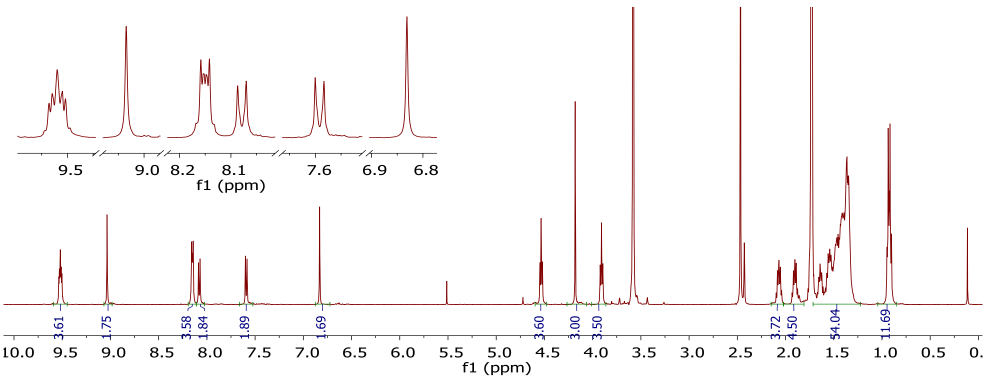

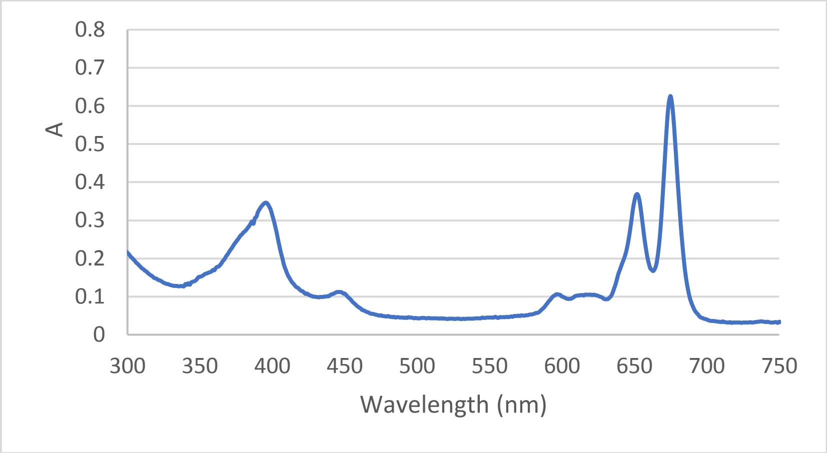

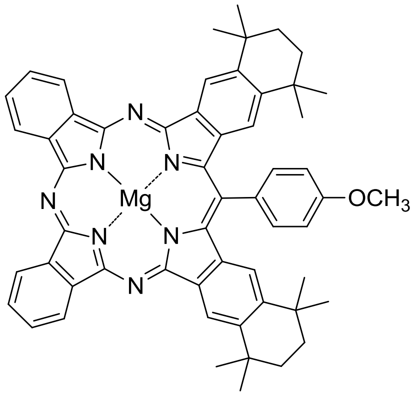

ABBA-TBTAP 20

Synthesised following the general synthesis of TBTAPs using phthalonitrile and aminoisoindolene **19**. This compound is isolated as the major product from the slow addition method. After chromatography, careful recrystallization from acetone and ethanol gave an analytically pure sample of the title compound as purple crystals (16 mg, 10%). **Chemical Formula:** $C_{56}H_{51}MgN_7O$. **M.p.** > 300°C. ^{1}**H NMR** (500 MHz, THF-d_8): δ = 9.57 (s, 2H),

9.53 (m, 4H), 8.18-8.11 (m, 4H), 8.08 (d, *J* = 8.4 Hz, 2H), 7.62 (d, *J* = 8.4 Hz, 2H), 7.39 (s, 2H), 4.21 (s, 3H, OCH_3), 1.81 (s, 8H), 1.37 (s, 24H). **^{13}C NMR** (125.7 MHz, THF-d_8): δ = 162.07, 157.31, 154.85, 152.82, 145.40, 144.62, 140.89, 140.81, 139.37, 139.02, 138.64, 134.59, 134.35, 129.45, 129.34, 127.04, 125.78, 124.79, 123.69, 120.98, 115.72, 56.40, 36.66, 36.64, 36.30, 36.16, 33.16, 33.13. **MS (MALDI-TOF):** *m/z* = 862.42 $[M]^+$ (100%). **UV-vis** (THF): λ_{max} (nm) (ε ($dm^3 \cdot mol^{-1} \cdot cm^{-1}$)) = 680 ($1.20 \cdot 10^5$), 656 ($6.56 \cdot 10^4$), 600 ($1.79 \cdot 10^4$), 449 ($1.65 \cdot 10^4$), 398 ($4.07 \cdot 10^4$).

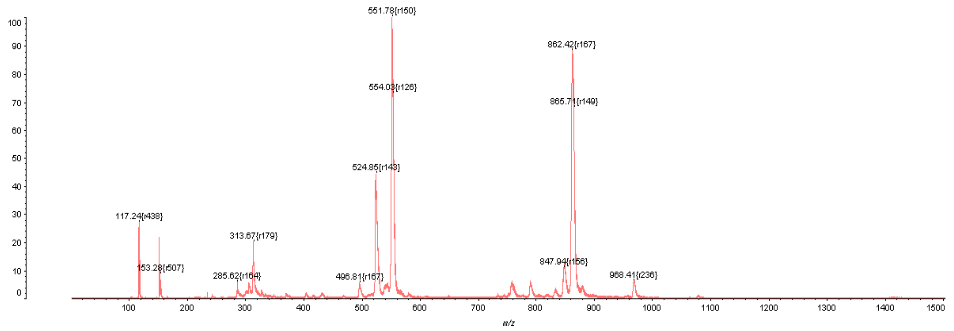

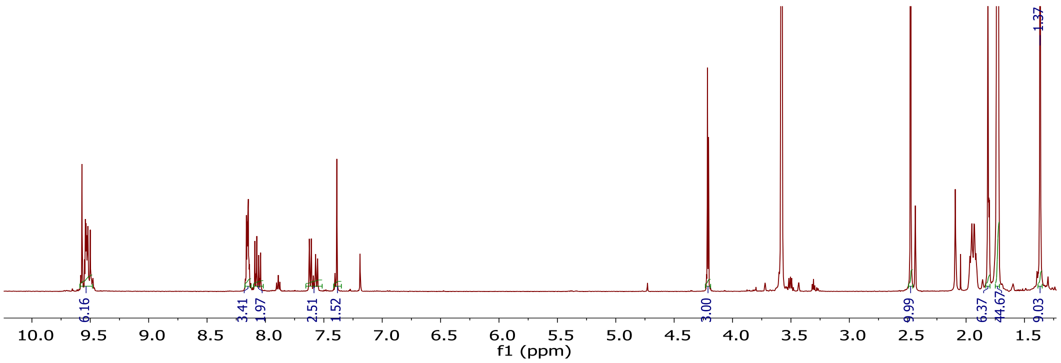

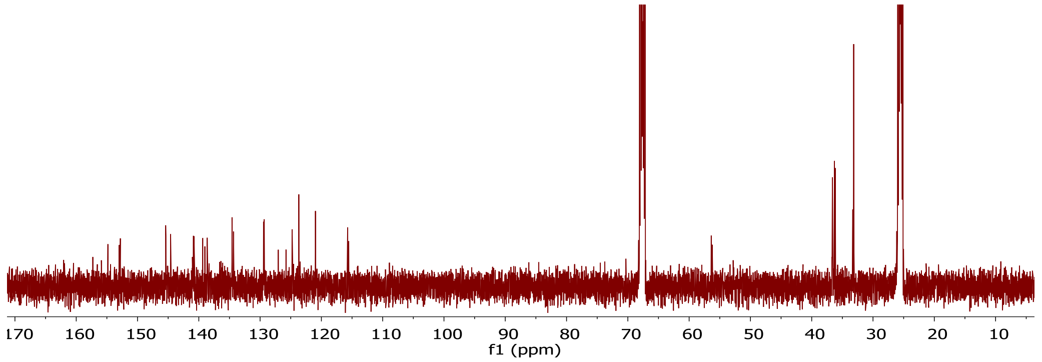

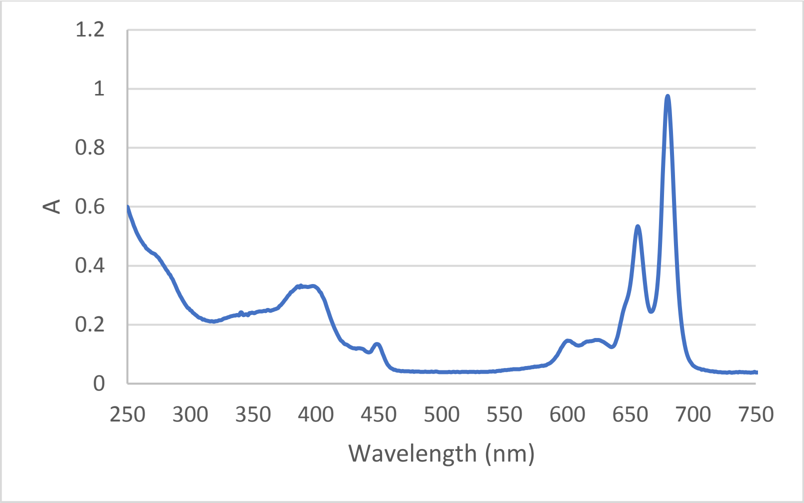

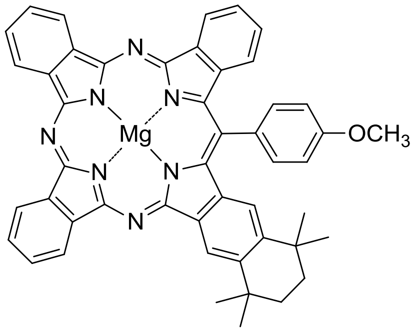

ABBB-TBTAP 21

Isolated from the above reaction alongside **20**. Recrystallisation from acetone and ethanol gave the title compound as purple crystals (5 mg, 2%). **Chemical Formula:** $C_{48}H_{37}MgN_7O$. **M.p.** > 300°C. **^{1}H NMR** (500 MHz, THF-d_8): δ = 9.60 (d, J = 7.5 Hz, 1H), 9.57 (s, 1H), 9.55-9.53 (m, 4H), 8.18-8.16 (m, 4H), 8.06 (d, J = 8.4 Hz, 2H), 7.92 (t, J = 6.9 Hz, 1H), 7.62 (t, J = 6.9 Hz, 1H), 7.57 (d, J = 8.4 Hz, 2H), 7.43 (d, J = 8.0 Hz, 1H), 7.21 (s, 1H), 4.21 (s, 3H, OCH_3), 1.80 (s, 4H), 1.37 (s, 12H). **^{13}C NMR** (125.7 MHz, THF-d_8): δ = 161.97, 155.84, 153.55, 152.87, 152.17, 145.59, 145.43, 144.58, 143.60, 141.16, 141.06, 140.84, 140.33, 139.47, 138.54, 136.28, 134.36, 129.76, 129.62, 129.49, 128.02, 127.32, 125.90, 125.61, 124.79, 123.78, 123.57, 120.99, 115.59, 56.24, 36.69, 36.61, 36.34, 36.16, 33.14, 33.11. **MS (MALDI-TOF):** m/z = 751.96 $[M]^+$ (100%). **UV-vis** (THF): λ_{max} (nm) (ε ($dm^3 \cdot mol^{-1} \cdot cm^{-1}$)) = 677 ($6.11 \cdot 10^4$), 653 ($3.61 \cdot 10^4$), 599 ($8.91 \cdot 10^3$), 447 ($8.91 \cdot 10^3$), 398 ($2.26 \cdot 10^4$).

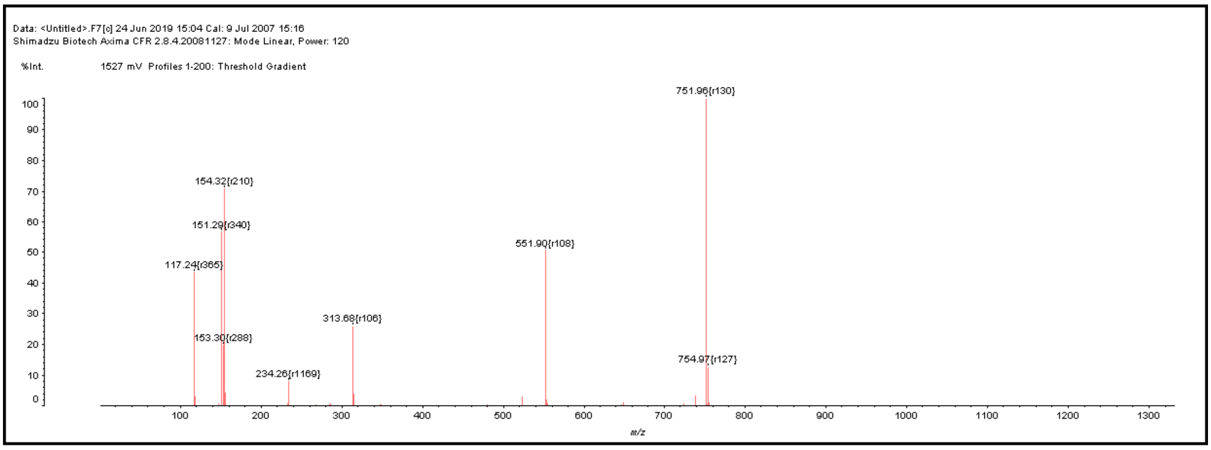

Data: <Untitled>.F7[c] 24 Jun 2019 15:04 Cal: 9 Jul 2007 15:16
Shimadzu Biotech Axima CFR 2.8.4.20081127: Mode Linear, Power: 120
%Int.
1527 mV Profiles 1-200: Threshold Gradient
751.96[r130]
154.32[r210]
151.29[r340]
551.90[r108]
117.24[r365]
313.68[r106]
153.30[r288]
754.97[r127]
234.26[r1169]
m/z

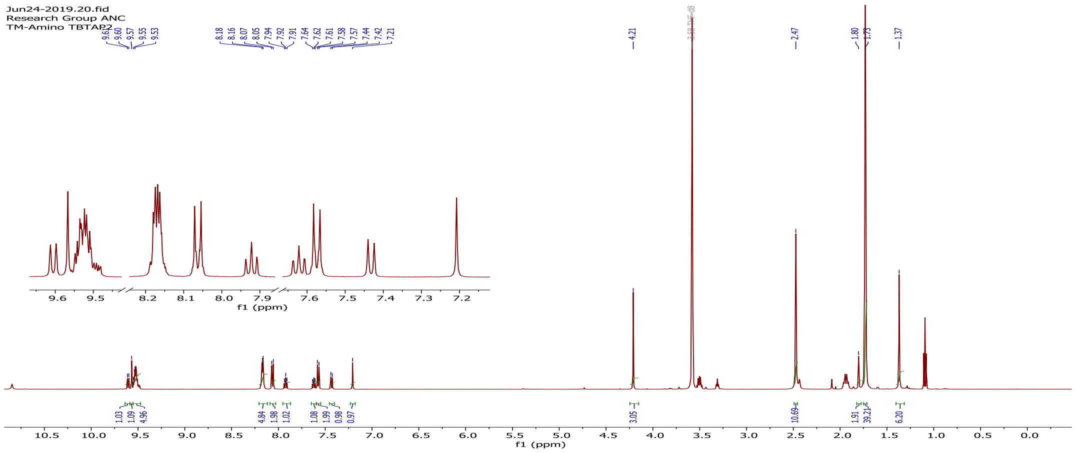

Jun24-2019.20.fid
Research Group ANC
f1 (ppm)
f1 (ppm)

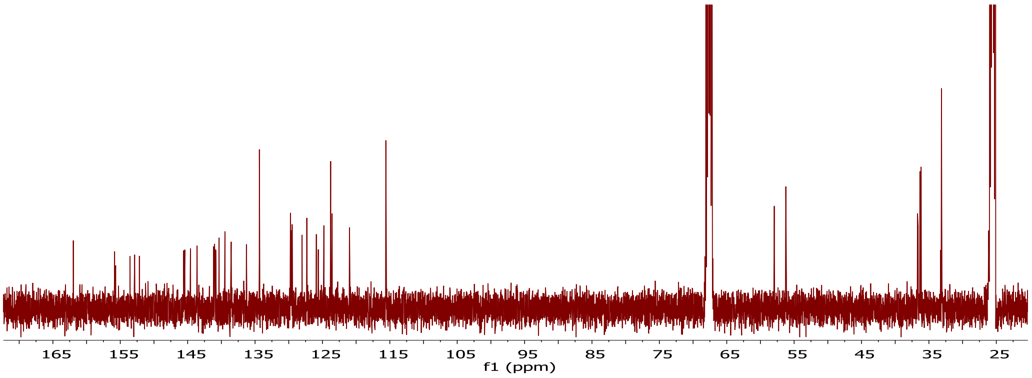

f1 (ppm)

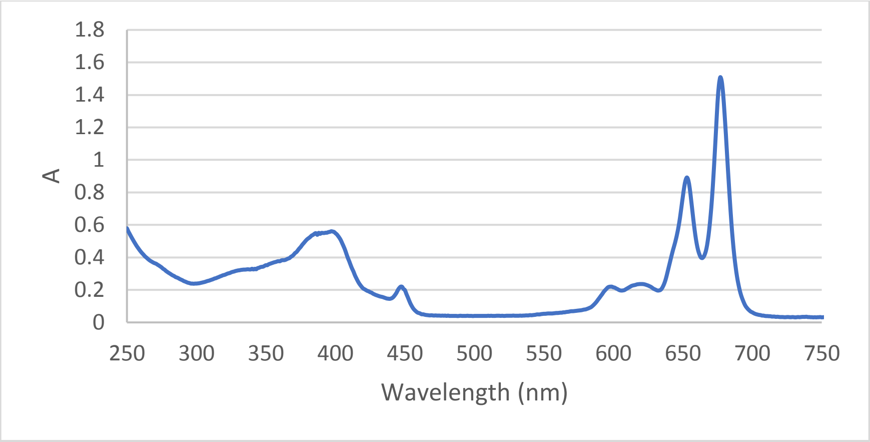
1.8
1.6
1.4
1.2
1
0.8
0.6
0.4
0.2
0
A
250
300
350
400
450
500
550
600
650
700
750
Wavelength (nm)

Crystallographic Details

Crystal structure analyses of compounds 7 and 8

General. Single bluish green plate-shaped crystals (**7** and **8**) were supplied. A suitable crystal 0.04×0.04×0.01 mm^3 (**7**) or 0.05×0.03×0.01 mm^3 (**8**) was selected and mounted on a MITIGEN holder in oil on a Rigaku FRE+ equipped with VHF Varimax confocal mirrors and an AFC12 goniometer and HG Saturn 724+ detector. The crystal was kept at a steady T = 100(2) K during data collection. The structure was solved with the **ShelXT**[15] structure solution program using the dual methods solution method and by using **Olex2**[16] as the graphical interface. The model was refined with version 2018/3 of **ShelXL**[17] using full matrix least squares minimisation on $\boldsymbol{F}^2$ minimisation. All non-hydrogen atoms were refined anisotropically. Hydrogen atom positions were calculated geometrically and refined using the riding model.

7. Solvent masking as implemented in **Olex2** was used to eliminate the electron contribution equivalent to 10 EtOH molecules per unit cell. Geometric equal distance and thermal restraints were applied to equivalent atom pairs in both disorder components of the Mg coordinated EtOH.

8. Geometric equal distance and thermal restraints were applied to equivalent atom pairs of all disorder components.

Compound 7

Crystal Data: $C_{66}H_{71}MgN_7O_2$, M_r = 1018.60, triclinic, P-1 (No. 2), a = 11.7079(3) Å, b = 14.0443(4) Å, c = 20.6637(5) Å, α = 103.510(2)°, β = 92.856(2)°, γ = 100.755(2)°, V = 3230.10(15) Å^3, T = 100(2) K, Z = 2, Z' = 1, μ(Mo K_α) = 0.072, 41059 reflections measured, 14741 unique (R_{int} = 0.0430) which were used in all calculations. The final wR_2 was 0.1649 (all data) and R_1 was 0.0611 (I≥2 σ(I)).

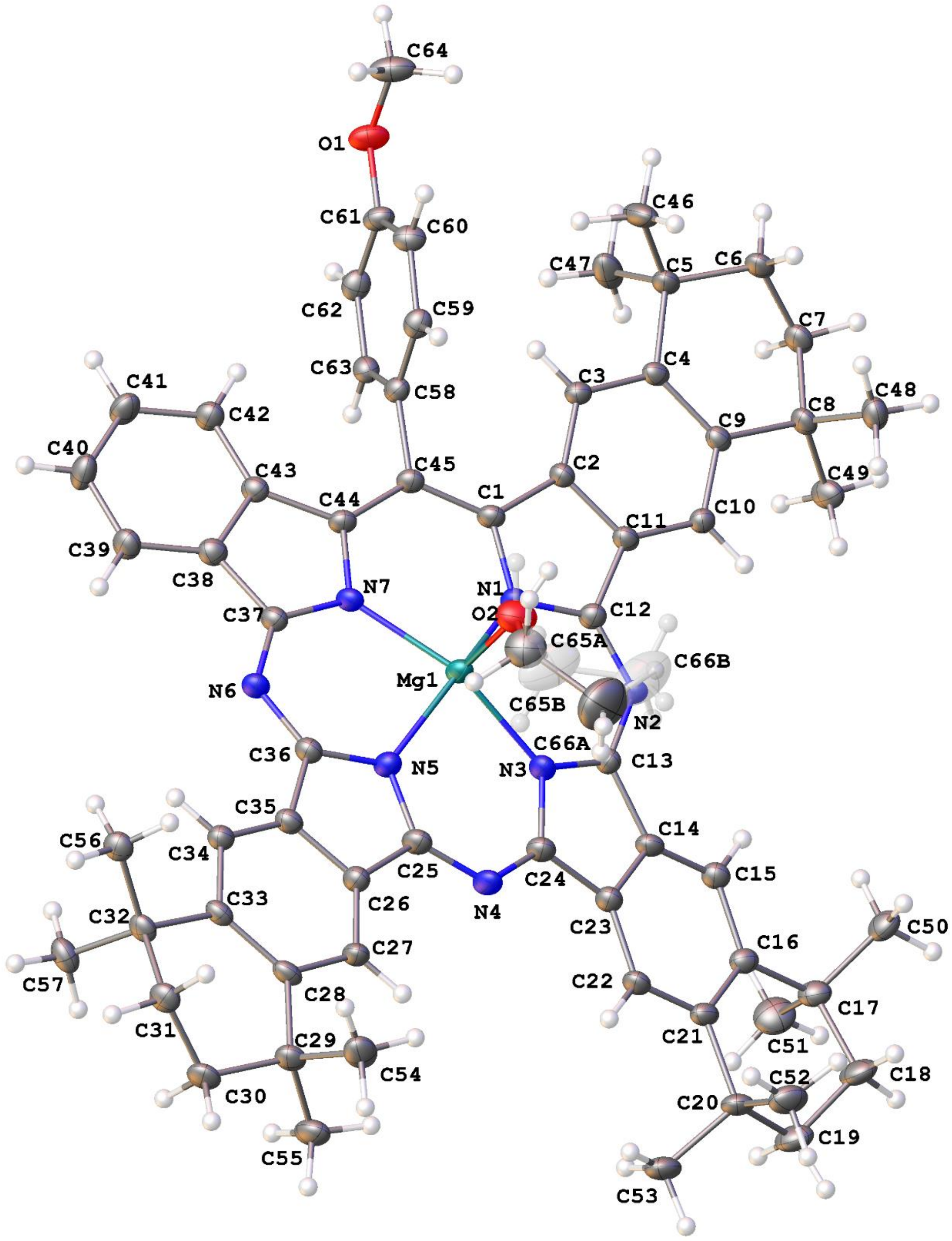

Compound 7: ADP ellipsoids shown at 50% probability. Minor component of disordered coordinated EtOH shown 'ghosted' for clarity.

Compound 8

Crystal Data: $C_{60}H_{63}MgN_7O_3$, M_r = 954.48, monoclinic, $P2_1/n$ (No. 14), a = 18.6220(5) Å, b = 10.3244(3) Å, c = 26.4965(8) Å, β = 91.971(3)°, $\alpha = \gamma$ = 90°, V = 5091.3(3) Å^3, T = 100(2) K, Z = 4, Z' = 1, μ(Mo K_α) = 0.089, 59130 reflections measured, 10395 unique (R_{int} = 0.0641) which were used in all calculations. The final wR_2 was 0.2148 (all data) and R_1 was 0.0741 (I≥2 σ(I)).

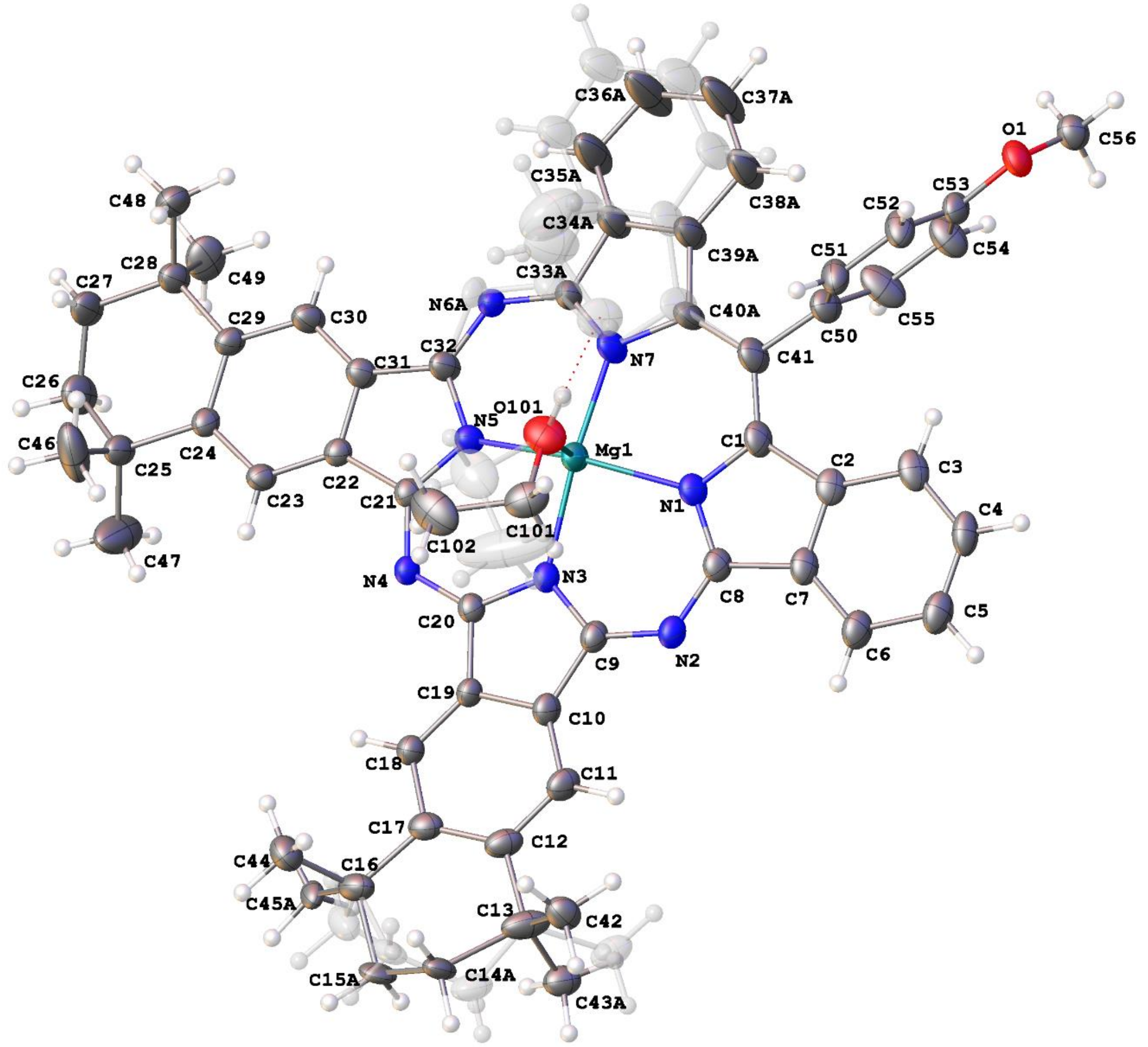

Compound 8: ADP ellipsoids shown at 50% probability. All minor disorder components and solvent EtOH shown 'ghosted' for clarity

Crystal structure analysis of 1-Br,2-C(NH$_2$)$_2$,4,5-(OMe)$_2$-benzene, Cl, H_2O, compound 16a

Crystal data: $C_9H_{12}BrN_2O_2$, Cl, H_2O, M = 313.58. Triclinic, space group P-1 (no. 2), a = 7.86437(16), b = 8.22233(16), c = 9.9670(2) Å, α = 78.3964(18), β = 76.7837(18), γ = 82.7698(16) °, V = 612.45(3) Å^3. Z = 2, Dc = 1.700 g cm^{-3}, F(000) = 316, T = 100.01(10) K, μ(Cu-Kα) = 65.7 cm^{-1}, λ(Cu-Kα) = 1.54184 Å.

The crystal was a colourless prism, *ca* 0.05 x 0.09 x 0.115 mm, mounted in oil on a small loop and fixed in the cold nitrogen stream on a Rigaku Oxford Diffraction XtaLAB Synergy diffractometer, equipped with Cu-Kα radiation, HyPix detector and mirror monochromator. Intensity data were measured by thin-slice ω-scans. Total no. of reflections recorded, to θ_{max} = 72.5°, was 16222 of which 2424 were unique (Rint = 0.032); 2422 were 'observed' with I > $2\sigma_I$.

Data were processed using the CrysAlisPro-CCD and -RED programs.[18] The structure was determined by the intrinsic phasing routines in the SHELXT[15] program and refined by full-matrix least-squares methods, on F^2's, in SHELXL.[17] The non-hydrogen atoms were refined with anisotropic thermal parameters. The phenyl ring hydrogen atoms were included in idealised positions and their Uiso values were set to ride on the Ueq values of the parent carbon atoms. The remaining hydrogen atoms were located in a difference map and were refined freely and isotropically. At the conclusion of the refinement, wR_2 = 0.054 and R_1 = 0.021 for all 2424 reflections weighted w = $[\sigma^2(F_o^2) + (0.0277\ P)^2 + 0.5283\ P]^{-1}$ with $P = (F_o^2 + 2F_c^2)/3$.

In the final difference map, the highest peak (*ca* 0.3 eÅ^{-3}) was near Br(1).

Notes on the structure

The principal moiety in this crystal is a 1-Br,2-C(NH$_2$)$_2$,4,5-(OMe)$_2$-benzene cation, with the positive charge distributed through the H_2N-C-NH$_2$ group; all the hydrogen atoms in this group were located in a difference map and were refined freely and isotropically. The C(2)-C(21) bond appears to be a normal single bond and the two C-N bonds are both rather short and equivalent at 1.312(2)Å.

The crystal contains, in addition to the cation, a chloride anion and a water molecule.

The bromine substituent, the two methoxy groups and the carbon atom of the diamino-methyl group, all lie on or very close to the plane of the phenyl ring. The C(NH$_2$)$_2$ group forms a separate plane, rotated 62.8(1)° from the phenyl group plane.

All the amino and water hydrogen atoms are involved in hydrogen bonds that link the moieties; the stronger bonds link the cations, through the chloride and water units, in paired chains (ladder-like) parallel to the *b*-axis, while weaker bonds, through the methoxy groups, connect the ladders to form hydrogen-bonded sheets.

The cations overlap in pairs about centres of symmetry; the two phenyl rings are *ca* 3.4 Å apart. Outside these pairs, there is off-set pairing about further symmetry centres so that C(1)-C(6) bonds are *ca* 3.6 Å apart.

Table S2. Hydrogen bonds in compound **16a**, in Ångstroms and degrees.

D-H...A	d(D-H)	d(H...A)	d(D...A)	<(DHA)
C(6)-H(6)...O(9)#2	0.93	2.50	3.419(2)	169.8
N(21)-H(21B)...Cl#3	0.86(3)	2.57(3)	3.3236(16)	147(2)
N(22)-H(22A)...O(9)	0.83(2)	1.97(3)	2.790(2)	170(2)
N(22)-H(22B)...Cl#3	0.90(3)	2.33(3)	3.1755(15)	156(2)
O(9)-H(91)...Cl	0.80(3)	2.38(3)	3.1696(14)	173(3)
O(9)-H(92)...Cl#4	0.81(3)	2.43(3)	3.2354(14)	173(2)

Symmetry transformations used to generate equivalent atoms:
#1 : x-1, y, z #2 : 1-x,1-y, 1-z #3 : 1-x, -y, 2-z
#4 : 1-x, 1-y, 2-z

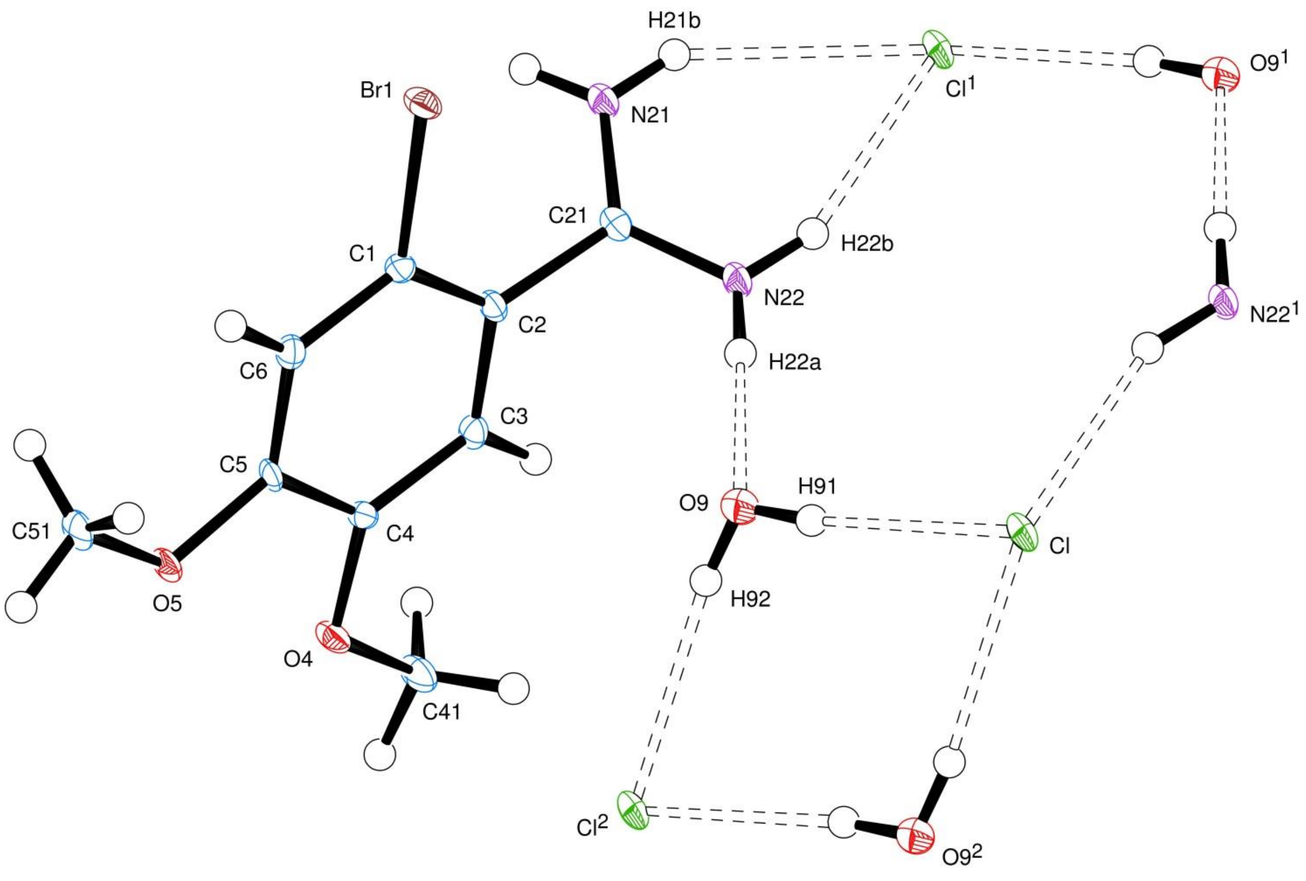

Compound 16a: View of a molecule of 1-Br,2-C(NH_2)$_2$,4,5-(OMe)$_2$-benzene, Cl, H_2O, compound **16a,** indicating the atom numbering scheme and the major hydrogen bonds. Thermal ellipsoids are drawn at the 50% probability level.

Crystal structure analysis of $(MeO)_2$-C_8H_2N-NH_2,=CH-C_6H_4-OMe, compound 17a

Crystal data: $C_{18}H_{18}N_2O_3$, M = 310.34. Orthorhombic, space group $Pna2_1$ (no. 33), a = 8.06974(5), b = 10.85362(6), c = 17.48441(9) Å, V = 1531.385(15) $Å^3$. Z = 4, Dc = 1.346 g cm^{-3}, F(000) = 656, T = 100.01(10) K, μ(Cu-Kα) = 7.54 cm^{-1}, λ(Cu-Kα) = 1.54184 Å.

The crystal was a yellow plate, *ca* 0.055 x 0.13 x 0.15 mm, mounted on a small loop and fixed in the cold nitrogen stream on a Rigaku Oxford Diffraction XtaLAB Synergy diffractometer, equipped with Cu-Kα radiation, HyPix detector and mirror monochromator. Intensity data were measured by thin-slice ω-scans. Total no. of reflections recorded, to θ_{max} = 72.5°, was 55632 of which 3033 were unique (Rint = 0.070); 3018 were 'observed' with $I > 2\sigma_I$.

Data were processed using the CrysAlisPro-CCD and -RED programs.[18] The structure was determined by the intrinsic phasing routines in the SHELXT[15] program and refined by full-matrix least-squares methods, on F^2's, in SHELXL.[17] The non-hydrogen atoms were refined with anisotropic thermal parameters. The hydrogen atoms on N(7) were located in a difference map and refined freely. The remaining hydrogen atoms were included in idealised positions and their Uiso values were set to ride on the Ueq values of the parent carbon atoms. At the conclusion of the refinement, wR_2 = 0.075 and R_1 = 0.028 (2B) for all 3033 reflections weighted $w = [\sigma^2(F_o^2) + (0.0491\ P)^2 + 0.2402\ P]^{-1}$ with $P = (F_o^2 + 2F_c^2)/3$. The absolute structure (Flack) parameter refined to 0.02(5), and the diagrams show the correct configuration.

In the final difference map, the highest peak (*ca* 0.2 e$Å^{-3}$) was near H(14b).

Notes on the structure

The molecule comprises two planar units, *viz* the isoindole nine-membered group and the phenyl ring of C(41-46). These rings are linked at C(10) and the torsion angle of C(9)-C(10)-C(11)-C(12) is 20.9(3)°. The three methoxy groups lie close to the plane of the adjoining aromatic rings.

Molecules are linked in chains parallel to the *a* axis through N(7)-H(7b)…N(8') hydrogen bonds. The second hydrogen of the N(7) amino group is not involved in any hydrogen bond formation but has a close contact, H(7a)…C(12) at 2.69 Å, on to the face of the phenyl ring. We note a 'weak hydrogen bond' between C(13)-H(13) and a neighbouring O(5) atom, where the H…O distance is 2.39 Å.

Table S4. Hydrogen bonds in compound **17a**, in Ångstroms and degrees.

D-H...A	d(D-H)	d(H...A)	d(D...A)	<(DHA)
N(7)-H(7B)...N(8)#1	0.89(3)	2.04(3)	2.920(2)	170(3)
C(13)-H(13)...O(5)#2	0.93	2.39	3.147(2)	138.2

Symmetry transformations used to generate equivalent atoms:
#1 : ½+x, ½-y, z #2 : 1-x, 1-y, ½+z

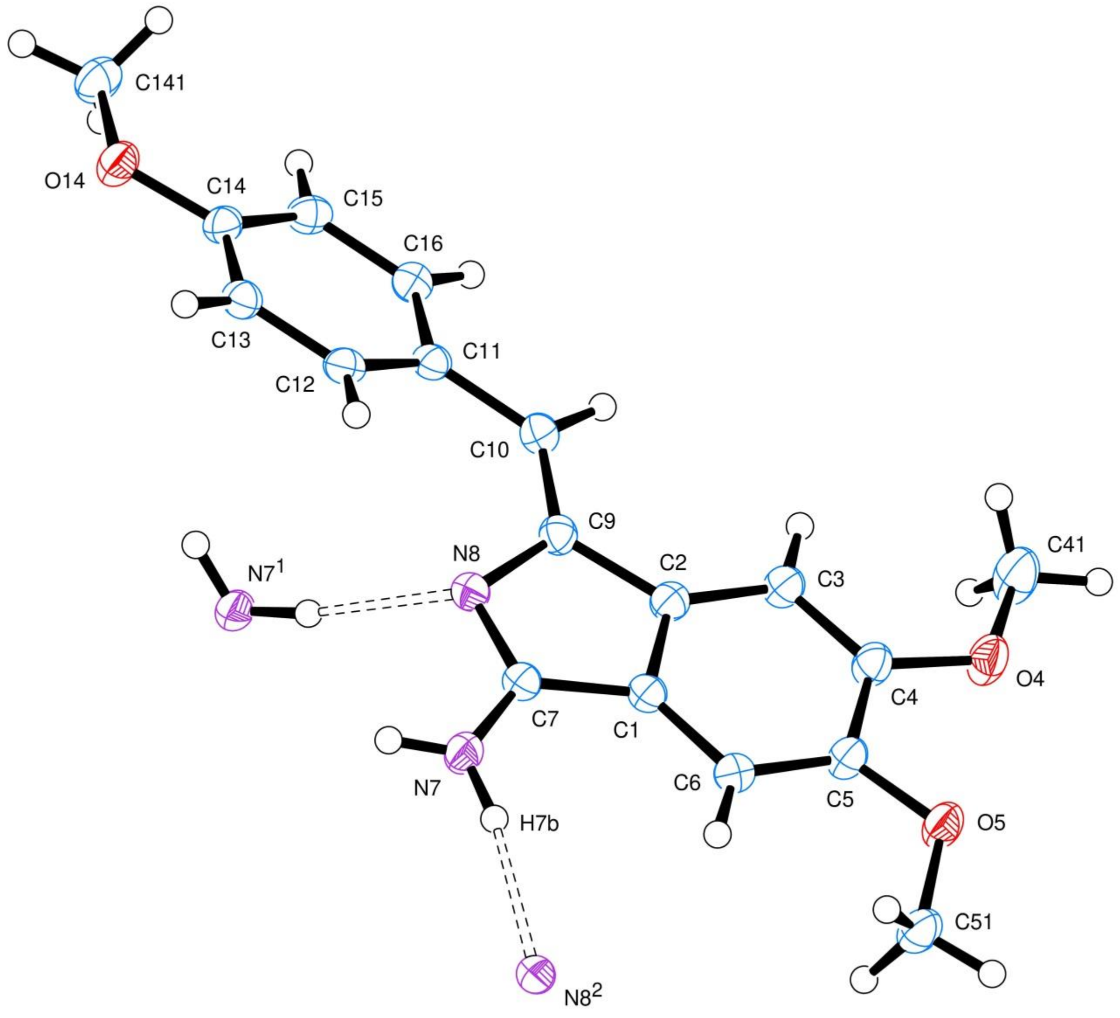

Compound 17a: View of a molecule of $(MeO)_2$-C_8H_2N-NH_2,=CH-C_6H_4-OMe, compound **17a**, indicating the atom numbering scheme. Thermal ellipsoids are drawn at the 50% probability level.

Crystal structure analysis of [Mg [porph-$(OMe)_4$,C-C_6H_4OMe)], EtOH], solvent, compound 18a

Crystal data: $C_{46}H_{37}MgN_7O_6$, *ca* $C_{0.6}$ $H_{1.75}$ O_2, M = 849.10. Orthorhombic, space group Pbca (no. 61), a = 18.7395(8), b = 16.9239(4), c = 25.4014(7) Å, V = 8055.9(5) $Å^3$. Z = 8, Dc = 1.400 g cm^{-3}, F(000) = 3547, T = 100.01(10) K, μ(Cu-Kα) = 9.40 cm^{-1}, λ(Cu-Kα) = 1.54184 Å.

The crystal was a dark red plate, *ca* 0.03 x 0.08 x 0.11 mm; it was mounted in oil on a small loop and fixed in the cold nitrogen stream on a Rigaku Oxford Diffraction XtaLAB Synergy diffractometer, equipped with Cu-Kα radiation, HyPix detector and mirror monochromator. Intensity data were measured by thin-slice ω-scans. Total no. of reflections recorded, to θ_{max} = 65°, was 207908 of which 6821 were unique (Rint = 0.097); 5675 were 'observed' with $I > 2\sigma_I$.

Data were processed using the CrysAlisPro-CCD and -RED programs.[18] The structure was determined by the intrinsic phasing routines in the SHELXT[15] program and refined by full-matrix least-squares methods, on F^2's, in SHELXL.[17] The structure comprises the magnesium complex and approximately two solvent (water) molecules. The non-hydrogen atoms were refined with anisotropic thermal parameters. Hydrogen atoms in the solvent region and located in difference maps were refined freely. The remaining hydrogen atoms were included in idealised positions and their Uiso values were set to ride on the Ueq values of the parent carbon atoms. At the conclusion of the refinement, wR_2 = 0.220 and R_1 = 0.094 (2B) for all 6821 reflections weighted $w = [\sigma^2(F_o^2) + (0.0923 P)^2 + 19.82 P]^{-1}$ with $P = (F_o^2 + 2F_c^2)/3$; for the 'observed' data only, R_1 = 0.083.

In the final difference map, the highest peak (*ca* 1.1 e$Å^{-3}$) was near H(10b) of the ethanol ligand.

Notes on the structure

The magnesium is coordinated at the centre of the porphyrin ligand, and removed 0.495(2) Å from the mean-plane of the four coordinating N atoms; it is also bound to an ethanol molecule, forming a square pyramidal pattern. This is all well-resolved and reliable. Then the hydroxyl group of the EtOH ligand is hydrogen bonded to an oxygen atom (probably) of a solvent/water molecule and that has further hydrogen bonds to a second water molecule which is linked by more hydrogen bonds to two further Mg-porphyrin molecules. There is some uncertainty and/or disorder about all the hydrogen atoms and the hydrogen bonds in this water/solvent region - I am assuming the solvent molecules are mostly water molecules, although there is one site of electron density here that refines best as 'half-a-carbon' atom, C(201), as part of a methanol molecule.

On the opposite side of the Mg-Porph system, a centrosymmetrically related molecule lies with the C(2-7) and C(26-31) rings of one molecule overlapping the C(26'-31') and C(2'-7') rings of the opposing molecule.

The methoxyphenyl group is linked to the porphyrin ring through the carbon atom C(33). The phenyl ring is almost perpendicular to the general porphyrin ring plane. H(3) and H(30), which are on adjacent benzo groups, have close H…π contacts with this ring; H(3) is 2.49 Å from C(43) and 2.55 Å from C(38), and H(30) is 2.57 Å from C(38). H(42) and H(43) of the phenyl ring are directed towards the underside of the centrosymmetrically related Mg-porph

group, with short H…π distances of H(42)…Mg’ at 3.06 Å, and … N(3’) at 2.82 Å, and H(43)…C(1’) at 2.56 Å and …N(1’) at 2.68 Å; we note also the short contact of H(44c)…C(16’) at 2.74 Å.

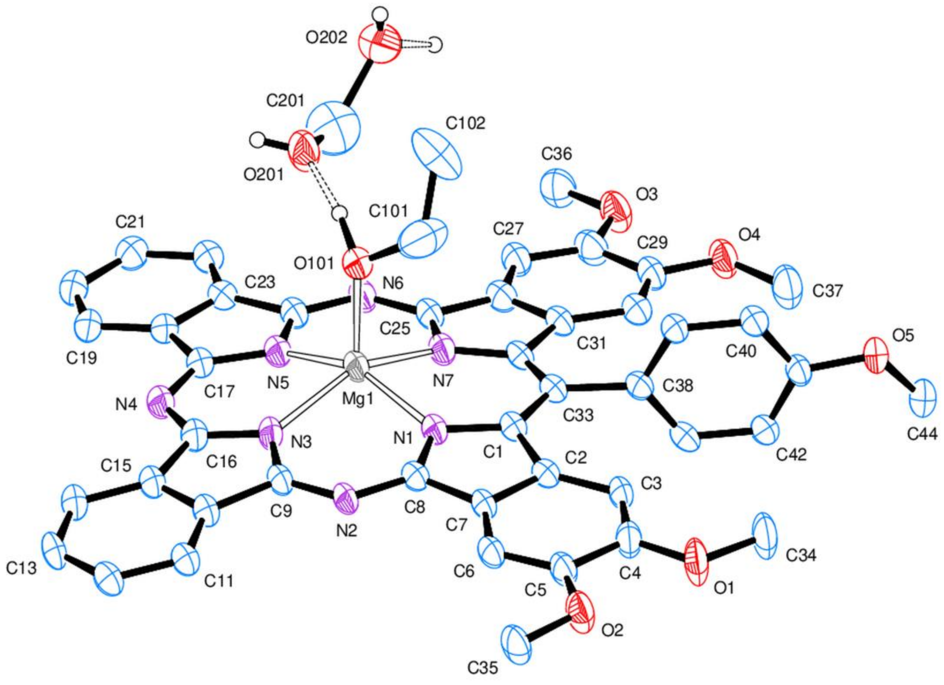

Compound 18a: View of a molecule of [Mg[porph-(OMe)$_4$,C-C_6H_4OMe)],OHEt], solvent, and the intermolecular hydrogen bonding, indicating the atom numbering scheme. Thermal ellipsoids are drawn at the 40% probability level.

For compounds 16a, 17a, 18a

Scattering factors for neutral atoms were taken from reference.[19] Computer programs used in this analysis have been noted above, and were run through WinGX[20] on a Dell Optiplex 780 PC at the University of East Anglia.

References

[1] a) C. E. Wagner, P. W. Jurutka, P. A. Marshall, T. L. Groy, A. van der Vaart, J. W. Ziller, J. K. Furmick, M. E. Graeber, E. Matro, B. V. Miguel, I. T. Tran, J. Kwon, J. N. Tedeschi, S. Moosavi, A. Danishyar, J. S. Philp, R. O. Khamees, J. N. Jackson, D. K. Grupe, S. L. Badshah, J. W. Hart, *J. Med. Chem.* **2009**, *52*, 5950-5966; b) N. Lamei, A. Foroumadi, S. Emami, M. Amini, A. Shafiee, *Chin. J. Chem.* **2010**, *28*, 1951-1956.

[2] H. A. Bruson, J. W. Kroeger, *J. Am. Chem. Soc.* **1940**, *62*, 36-44.

[3] I. Rose, C. G. Bezzu, M. Carta, B. Comesaña-Gándara, E. Lasseuguette, M. C. Ferrari, P. Bernardo, G. Clarizia, A. Fuoco, J. C. Jansen, K. E. Hart, T. P. Liyana-Arachchi, C. M. Colina, N. B. McKeown, *Nat. Mater.* **2017**, *16*, 932-937.

[4] a) T. Wenderski, K. M. Light, D. Ogrin, S. G. Bott, C. J. Harlan, *Tetrahedron Lett.* **2004**, *45*, 6851-6853; b) Y. B. Platonova, A. N. Volov, L. G. Tomilova, *J. Catal.* **2019**, *373*, 222-227.

[5] A. N. Cammidge, H. Gopee, *J. Mater. Chem.* **2001**, *11*, 2773-2783.

[6] a) T. Sauer, G. Wegner, *Mol. Cryst. Liq. Cryst. Inc. Nonlinear Opt.* **1988**, *162*, 97-118; b) J. Sleven, C. Görller-Walrand, K. Binnemans, *Mater. Sci. Eng. C* **2001**, *18*, 229-238.

[7] G. P. Ellis, T. M. Romney-Alexander, *Chem. Rev.* **1987**, *87*, 779-794.

[8] S. A. Mikhalenko, L. I. Solov'eva, E. A. Luk'yanets, *J. Gen. Chem. USSR* **1988**, *58*, 2618–2619.

[9] J. Metz, O. Schneider, M. Hanack, *Inorg. Chem.* **1984**, *23*, 1065-1071.

[10] L. R. Donaldson, S. Wallace, D. Haigh, E. E. Patton, A. N. Hulme, *Org. Biomol. Chem.* **2011**, *9*, 2233-2239.

[11] A. I. Koptyaev, N. E. Galanin, G. P. Shaposhnikov, *Russ. J. Gen. Chem.* **2016**, *86*, 854-858.

[12] S. Dalai, V. N. Belov, S. Nizamov, K. Rauch, D. Finsinger, A. de Meijere, *Eur. J. Org. Chem.* **2006**, *2006*, 2753-2765.

[13] M. Hellal, G. D. Cuny, *Tetrahedron Lett.* **2011**, *52*, 5508-5511.

[14] A. Díaz-Moscoso, G. J. Tizzard, S. J. Coles, A. N. Cammidge, *Angew. Chem. Int. Ed.* **2013**, *52*, 10784-10787.

[15] G. M. Sheldrick, SHELXT, Program for crystal structure determination, *Acta Cryst. 71*, 3-8, **2015**.

[16] O. V. Dolomanov, L. J. Bourhis, R. J. Gildea, J. A. K. Howard, H. Puschmann, *J. Appl. Cryst.* **2009**, *42*, 339-341.

[17] G. M. Sheldrick, SHELXL, Program for crystal structure refinement, *Acta Cryst. 64*, 112-122, **2008**.

[18] CrysAlisPro, Program for the solution of crystal structures, Rigaku Oxford Diffraction Ltd., Abingdon (UK), **2018**.

[19] *International tables for x-ray crystallography, Vol. C*, Kluwer Academic Publishers, Dordrecht (The Netherlands), **1992**.

[20] L. Farrugia, *J. Appl. Cryst.* **2012**, *45*, 849-854.
